# Modelling of paratuberculosis spread between dairy cattle farms at a regional scale

**DOI:** 10.1186/s13567-015-0247-3

**Published:** 2015-09-25

**Authors:** Gaël Beaunée, Elisabeta Vergu, Pauline Ezanno

**Affiliations:** INRA, UR1404 Unité Mathématiques et Informatique Appliquées du Génome à l’Environnement (MaIAGE), F-78352 Jouy-en-Josas Cedex, France; INRA, LUNAM Université, Oniris, UMR1300 BioEpAR, CS40706, F-44307 Nantes, France

## Abstract

*Mycobacterium avium* subsp. *paratuberculosis* (*Map*) causes Johne’s disease, with large economic consequences for dairy cattle producers worldwide. *Map* spread between farms is mainly due to animal movements. Locally, herd size and management are expected to influence infection dynamics. To provide a better understanding of *Map* spread between dairy cattle farms at a regional scale, we describe the first spatio-temporal model accounting simultaneously for population and infection dynamics and indirect local transmission within dairy farms, and between-farm transmission through animal trade. This model is applied to Brittany, a French region characterized by a high density of dairy cattle, based on data on animal trade, herd size and farm management (birth, death, renewal, and culling) from 2005 to 2013 for 12 857 dairy farms. In all simulated scenarios, *Map* infection highly persisted at the metapopulation scale. The characteristics of initially infected farms strongly impacted the regional *Map* spread. Network-related features of incident farms influenced their ability to contaminate disease-free farms. At the herd level, we highlighted a balanced effect of the number of animals purchased: when large, it led to a high probability of farm infection but to a low persistence. This effect was reduced when prevalence in initially infected farms increased. Implications of our findings in the current enzootic situation are that the risk of infection quickly becomes high for farms buying more than three animals per year. Even in regions with a low proportion of infected farms, *Map* spread will not fade out spontaneously without the use of effective control strategies.

**Electronic supplementary material:**

The online version of this article (doi:10.1186/s13567-015-0247-3) contains supplementary material, which is available to authorized users.

## Introduction

Understanding how the contact structure between individuals or populations affects the spread and persistence of infectious human and animal diseases is of great importance for better controlling their spread [1,2]. Pathogens can propagate among populations of hosts through various transmission routes. Movements of infected hosts represent a major pathway [3]. Indeed, these movements directly affect the epidemiological status of destination populations. Moreover, they can relate distant populations influencing disease spread at a large scale [4].

In Europe, due to regulation following the bovine spongiform encephalopathy crisis, national databases have been set up with the objective to exhaustively trace cattle movements between farms. Such data were largely investigated using methodological tools from network analysis [5-9]. In particular, their temporal variability has been shown to be a key determinant in the assessment of their vulnerability to infection emergence and propagation [5,10]. As information on animal movements between farms is now available over several years in many countries [11,12], it can be used as underlying structure of pathogen spread between cattle farms, when investigating regional dynamics [13].

Paratuberculosis, or Johne’s disease, is considered as mainly introduced into farms by purchasing infected stock [14]. This slow progressive disease observed worldwide [15,16] is due to *Mycobacterium avium* subsp. *paratuberculosis* (*Map*). It is one of the most important enzootic infectious diseases in dairy cattle with a large economic impact for producers due to decreased milk production, premature culling, reduced slaughter value, low fertility, and increased animal replacement rate [17,18]. Infection usually occurs in the first year of life [19], newborns being the most susceptible animals. Transmission occurs in utero [20] and through the ingestion of *Map* via contaminated colostrum, milk or faeces [21]. The progression of animals through the different *Map* infection states is a complex continuous process with intermittence in shedding and a late onset of clinical signs. Because of the low sensitivity of diagnostic tests currently available, especially for the early stages of the disease, *Map* spread at a regional scale cannot be easily observed and remains poorly understood. Hence, it is not straightforward to evaluate and compare the efficiency of control measures through field studies, which are, besides, long and expensive. In this context, modelling provides relevant and complementary insights for the study of paratuberculosis progression at a regional scale.

For slowly progressive diseases, such as paratuberculosis, local patterns of infection spread vary over time and are often heterogeneous among populations. Hence, infection dynamics within populations need to be accounted for when exploring the transmission of such diseases at a metapopulation scale (e.g. for tuberculosis in cattle [13]). Moreover, livestock populations are managed by farmers, leading to a short life expectancy of animals, a large renewal rate, and a well-characterized within-herd structure of contacts. Herd size and farm management also vary among farms. Since all of these factors largely impact pathogen spread within and between populations, they also should be considered to adequately represent and better understand pathogen spread through multi-level approaches, from local to regional scales.

Several models of *Map* spread within a cattle farm have been proposed (most of them reviewed in [22]) to test various hypotheses on transmission pathways [23,24], to investigate economic consequences of the disease [25-27], and to compare control strategies at the farm level [28-30]. It has been shown that the two main transmission routes within a farm are the indirect transmission through the farm environment contaminated by infectious adults and the vertical in utero transmission. [31] Moreover, the large influence of the farm management on *Map* spread has been evidenced [30]. At a regional scale, fewer approaches have been proposed for paratuberculosis [32,33], none accounting for the within-farm indirect *Map* transmission in relation with farm management.

To better understand the main features of *Map* spread at a regional scale in a metapopulation of dairy cattle, we developed a multi-scale modelling framework. *Map* epidemiological models defined at the farm scale are coupled through animal trade movements. Farm management is also considered. The model is generic, but in this study it was calibrated to be in agreement with farming systems and herd demography as observed in Brittany, a region in Northwestern France. Three main features were explored through intensive simulations. We evaluated the influence of the characteristics of initially infected farms on the regional *Map* spread and persistence over almost a decade. We characterized farm profiles at risk to receive or transmit the disease. We also studied the within-farm infection dynamics, namely the probability of extinction and the prevalence, in the context of a pathogen circulating between connected populations (i.e. a metapopulation).

## Materials and methods

### Modeling *Map* spread at a regional scale

The regional discrete-time model of *Map* spread consists in coupling numerous (one per farm) stochastic within-farm epidemiological models through cattle trade movements. Connected dairy farms located in a given region are characterized by their size and population dynamics in relation with their management (births, deaths, culling, and renewal processes). For both animal movements and farming management, real observed data are plugged into the model.

#### Within-farm model of Map spread

We adapted the model of *Map* spread within a structured dairy cattle farm described by Marcé et al. [30,31], as it includes most of the current knowledge on the mechanisms involved in this infection.

**Figure 1:**
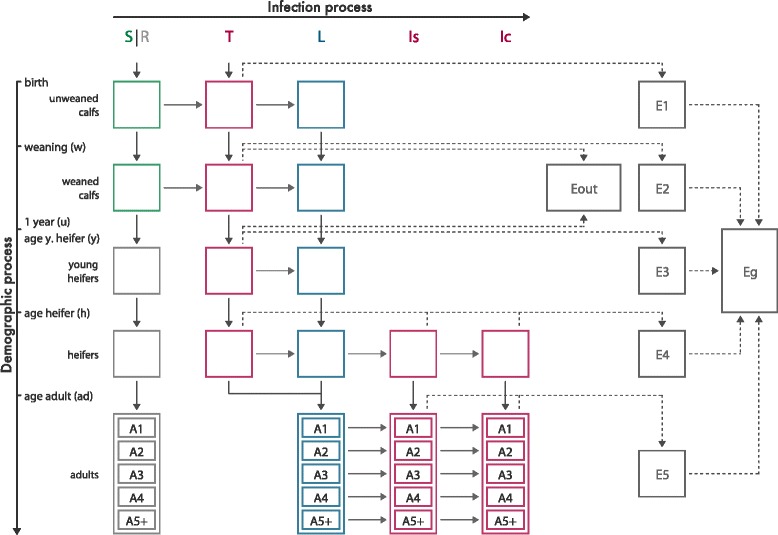
**Conceptual model of Map spread in a dairy cattle farm.** S, susceptible; R, resistant; T, transiently infectious; L, latently infected; Is, Ic, infectious and subclinically infected vs. clinically affected animals, respectively. Ei, indoor environment in housing *i*, with *i* in {1,…,5}(depends on age and season); Eg, general environment of the farm; Eout, outdoor environment of grazing calves; Aj, cows in adult group *j*, with *j* in {1,…,5}. Contributions to the environment contamination are represented by dotted lines. Exit rates from each compartment are not represented (adapted from Marcé et al. [30]).

The model and its assumptions are described in details in [31] In brief, this model is a stochastic compartmental model in discrete time (with a time step of one week) that jointly describes population and infection dynamics. Since *Map*-infected individuals exhibit slow progression through health states, the fixed time-step of one week, smaller than the average time interval between two events, was satisfactory. The herd is structured into five age groups and animals are distributed into six infection states (Figure 1): susceptible (*S*) before 1 year of age, resistant (*R*) at older ages, transiently infectious (*T*) just after the infection, latently infected but not infectious (*L*), infectious without symptoms (*I*_*S*_), clinically affected and highly infectious (*I*_*C*_). The model accounts for the decrease in susceptibility to infection with age (exponential decay). Infection of animals older than one year of age was neglected in the model (all these animals are in *R* compartment), since it is very rare in the field [19,34] and has been observed mostly during experiments with oral or intravenous inoculation of large doses of pathogen. The model also accounts for the heterogeneity in shedding among infectious animals, both between infection states and between animals in the same state. After an initial phase of shedding observed just after infection (state *T*), shedding barely can be observed before the first calving [35,36] and therefore is neglected (animals do not shed in state *L*). Five transmission routes are taken into account: in utero transmission and four indirect transmission pathways, since *Map* is able to survive in the environment. Indirect transmission can occur through the ingestion of contaminated milk, colostrum, and faeces, the latter arising either from the calf farming environment contaminated by shedding calves, or from the general farm environment contaminated by shedding adults. Six contaminated farm environments (*E*_*i*_) are modelled, one per age group and one for the general farm environment. The diagram flow of the model is represented in Figure 1.

Starting from this model, we made some simplifications and adaptations. First, the analysis of the model revealed that raising calves in individual pens during a few weeks hardly had any effect on *Map* spread as long as the separation from adults was not perfect [30]. The within-farm model is simplified accordingly, calves being assumed to be managed in collective pens and to be exposed to the associated environment since birth. Moreover, calf-to-calf transmission was identified as a minor route of transmission [31]. As male calves are generally sold a few weeks after birth, they are no longer considered. Second, in [31] herd size was kept stable by the sale of heifers and culling of cows using density-dependent processes. We modified the model so that animal movements (following purchase and sale) are deterministically incorporated from data on animal trade. Birth events also are plugged deterministically from data. Mortality and culling rates are still stochastic processes but with parameters calibrated from observed data, specifically for each farm.

In addition to all the simplifications mentioned above, the new version of the model, recoded in C++, is computationally much more efficient and modular, which renders it optimal for incorporation into a larger metapopulation model. All the variables and equations describing the within-farm dynamics are detailed in Additional file 1, section A.

#### Regional model of Map spread accounting for between-farm animal movements

In the regional model, date of movements, origin and destination farms, and age of traded animals are recorded in the database and hence deterministically implemented. The health state of every traded animal is randomly selected according to the prevalence of infection in the source farm at the time of movement. Every animal can be selected for a movement, except those with clinical signs (*Ic*). The health state *X*_*i* → *j*_^*a*,*k*^ of an animal *k* among *N*_*i* → *j*_^*a*^ animals of age *a* moving from farm *i* to farm *j* is drawn from a multinomial distribution. This writes as: *X*_*i* → *j*_^*a*,*k*^ ∼ *Multinomial*(1, [*p*_*i*_^*a*,*S*/*R*^, *p*_*i*_^*a*,*T*^, *p*_*i*_^*a*,*L*^, *p*_*i*_^*a*,*Is*^]), with ∑_*Z* ∈ (*S*/*R*,*T*,*L*,*Is*)_*p*_*i*_^*a*,*Z*^ = 1 for all *a, i* and *j*, where *p*_*i*_^*a*,*Z*^ represents the proportion of animals of age *a*, in health state *Z*, in farm *i*. These proportions are specifically calculated at the time of movement occurrence (for reasons of simplifications, time is omitted in the equation above).

In the unlikely case where there is no animal of the right age in the model as observed in the data, an animal is selected in the closest age group. In the case data specifies that an animal is purchased from outside Brittany, its health state is determined again using the previous equation, but the probabilities *p*^*a*,*Z*^ are calculated on the whole metapopulation considered, at the time of movement and for the corresponding age. The underlying assumption is that the risk of introduction of an infected animal is the same from outside as from within the metapopulation.

### Animal trade data and network representation

Information on animal movements was extracted from the French cattle identification database (FCID), for the period from 2005 to 2013 (nine years). This database records the life history of all cattle animals from birth to death, including movements between holdings (i.e. farms, markets, and assembling centres). For each animal, the information concerns its country code and national identification number, breed, date and farm of birth, sex, as well as all the holdings to which it belonged during its life time, the cause and date of entry into each holding (birth, purchase), the cause and date of exit from each holding (death, sale). Based on this information, we built the trade network formed by cattle movements among holdings, underlying the metapopulation contact structure. As the time spent by animals in markets and assembling centres is rather short (less than one day in markets and less than several days in assembly centres) and thus expected not to give rise to new infections, we rebuilt the trade network by replacing indirect farm-to-farm connections (passing through markets and assembling centres) by direct farm-to-farm connections. Hence, in the resulting network, farms represent the nodes and their trade relationships define the links. This network is directed (trade is not symmetric), weighted (the number of animals exchanged varies among pairs of farms) and time-varying (animal transactions occur at specific times).

Network attributes of a given node can inform on the node contribution, relatively to other nodes, regarding the ability of pathogens to invade and keep spreading and the epidemic burden following this invasion (both at local and metapopulations levels). Two key characteristics of node connectivity are used: degree and strength. The in-degree (out-degree) of a node is defined as the number of incoming (outgoing) links. The in-strength is defined as the number of animals purchased (incoming movements) from other nodes, whereas the out-strength is the number of animals sold (outgoing movements) to other nodes. From these attributes, the polarity of each node can be defined as the difference between its in-strength and out-strength over their sum (Moslonka-Lefebvre M, Gilligan C, Monod H, Belloc C, Ezanno P, Filipe J, Vergu E: Market analyses of livestock trade networks to inform the prevention of joint economic and epidemiological risk, submitted). By construction, this indicator takes its values between -1 and 1. Nodes with negative polarity less than -0.25 were labelled as “rather sellers”, whereas those with positive polarity greater than 0.25 as “rather buyers”, and those with polarity between -0.25 and 0.25 as “wholesalers”.

**Figure 2:**
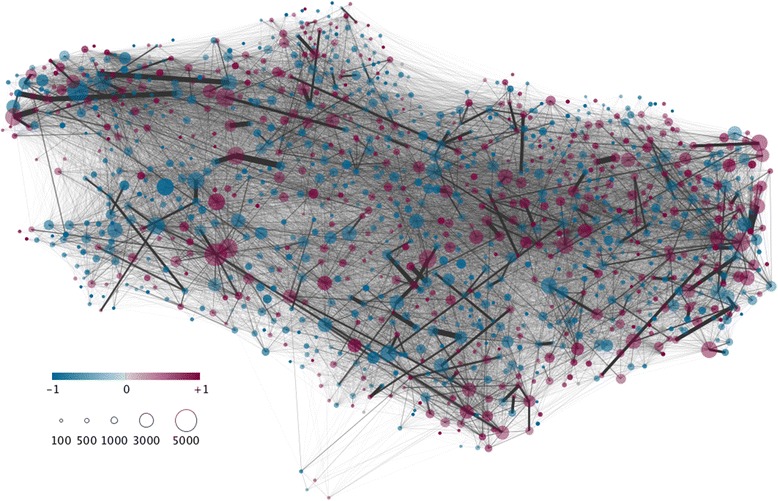
**Network representation of cattle trade data in Brittany (Northwestern France) between dairy farms from 2009 to 2013.** Diagram shows animal movement data aggregated spatially by municipality and temporally over the whole period. Size of filled circles corresponds to the number of animals (yearly average herd size) present in each municipality (represented on the map at its geographical location), and their colour to the polarity (blue when rather seller and red when rather buyer). Lines represent animal movements between municipalities (direction is neglected), and their thickness is proportional to the number of traded animals. Movements from and to outside the metapopulation are not shown.

We focused our study on dairy cattle farms located in Brittany, in Northwestern France. This region is characterized by a high density of dairy cattle (85% of cows are dairy cows) [37]. Farms were selected according to their type and size, only those having more than 15 dairy females being included in the network. Such farms are assumed to be professional ones with a dairy production unit. French dairy cattle herds are mainly composed of females, breeding being based on artificial inseminations. Therefore, only movements of females of dairy or crossed breed are considered in the network, neglecting fattening activities that are most often conducted in a different building or area of the farm. The resulting metapopulation is made of 12 857 farms, which tend to be rather sellers (72.7%) than buyers (26.2%). The network (Figure 2, aggregated over 2009–2013 for illustration) is composed of 919 304 animal movements over the observed period (2005–2013), among which 223 968 movements are between farms in the metapopulation, the others being from and to external holdings. The in- and out-degree distributions are highly right skewed, the majority of farms making relatively few contacts over the period considered (see Additional file 2). The exchanged animals are mainly young ones (39.5% before weaning) and lactating cows (37.4% older than 2.5 years of age).

### Parameter values, simulated scenarios, model outputs and simulations analysis

#### Model parameterisation

Parameter values of the within-farm model related to epidemic dynamics are identical to those presented in [31] (reported in the Additional file 1, section C). Parameters related to population dynamics, such as herd size and culling rates are calibrated on data, specifically for each farm of the metapopulation (see Additional file 1, section B, for distributions of these parameters). The agreement between observed and predicted herd size over the 9-year period was considered acceptable if there was at most 20% of gap between average predictions and observations for at least seven years among nine. Animal movements between farms completely match observed trade exchanges between farms (data described above).

#### Simulated scenarios

To assess the impact of the initial contamination severity at both metapopulation and farm levels, several scenarios were simulated by varying three criteria related to the initial conditions. We tested two values for the proportion of initially infected farms (1 and 10%) and four average levels (and related distributions) for the initial within-herd prevalence of infection within infected farms (A: very low, B: low, C: medium, D: high). These latter distributions were obtained by simulating the infection dynamics in isolated primarily infected farms during 1, 4, 7, and 10 years previous to any connection in the metapopulation. To evaluate how farms’ characteristics impact *Map* regional spread, primarily infected farms were chosen using three different options: (1) uniform random choice among farms selling at least one animal to another farm of the metapopulation during the period considered; (2) random choice weighted by farm out-degree, farms with large out-degree being preferentially selected; (3) random choice weighted by farm out-strength, farms with large out-strength being preferentially selected. For options 2 (respectively 3), farms were selected according to a discrete distribution where each farm *i* (among *n*) has the following probability of being chosen: $P\left(i\Big|{w}_1,{w}_2,\dots, {w}_n\right)=\frac{w_i}{{\displaystyle {\sum}_{k=1}^n}{w}_k},\left(1\le i\le n\right),$ where *w*_*i*_ is the out-degrees (respectively out-strength) of farm *i*.

Combining these three criteria led to 24 scenarios (2 × 4 × 3). Outputs were calculated based on 1000 runs per scenario, over the period 2005–2013.

#### Model outputs

The model behaviour was analysed using two kinds of outputs, specifically calculated for each scenario tested. First, we analysed *Map* spread at the metapopulation scale. For each scenario, we evaluated the probability of *Map* persistence in the metapopulation, defined at a given time point as the proportion of runs for which *Map* was still present in the metapopulation (in at least one farm). Then, among replications showing a persistent infection at the end of the simulated period, we evaluated the median and the empirical confidence interval based on percentiles (percentile 10 – percentile 90) of the proportion of infected farms in the metapopulation over time. Second, we investigated *Map* spread at the farm scale. We defined the probability for a farm of acquiring infection as the proportion of runs for which it has been infected at least once over the period. We counted the number of initially *Map*-free farms that have been infected per incident farm (i.e. tertiary cases caused by initially *Map*-free farms that have been infected, becoming secondary cases). This latter output enabled us to identify which farms were the most at-risk of spreading *Map* in the initial stage of the regional disease spread apart from prevalent farms (i.e. initial cases). We defined the probability of *Map* persistence in incident farms after a 5-year period as the ratio between the number of incident farms constantly infected during the 5 years after their infection set up and the total number of incident farms. A farm that has been infected, where infection has fade out, and that has been infected again was counted twice as an incident farm. We investigated the distribution of the within-herd prevalence of infection 5 years after the time of individual infection in the subpopulation of already infected farms. These two latter outputs were appropriate to assess, for a given farm, the impact on the within-farm infection dynamics of having connections with other farms in a region where *Map* propagates, compared to being isolated.

#### Simulations analysis

Variations in farm-level outputs were analysed with respect to the number of infected animals purchased and the farm characteristics. Farm-related outputs investigated were: prevalence in infected animals, infection duration, probability of infection and probability of persistence. Herd size and farm characteristics related to the connectivity on the network, such as in- and out-degrees, in- and out-strengths, and polarity were the characteristics considered. Distributions of these characteristics in different subpopulations were compared using chi-square tests. In order to identify determinants of the probability of infection of *Map*-free farms, general linear models including first one explanatory variable among herd size, degree (in and out), strength (in and out) and polarity, and then all variables, were tested. We used Akaike information criterion (AIC) and adjusted McFadden’s pseudo R^2^ to evidence the best model. Analyses were performed using the glm function (with binomial link and logit transformation) and BaylorEdPsych package (for model selection criteria) of R-software [38].

## Results

Preliminary explorations of disease-free population dynamics showed a good agreement between simulated and observed data. Demographic trends were adequately reproduced for 99% of the farms according to the empirical criterion defined, comparing observed and predicted herd sizes over time. The model was able to track changes in herd size in most of the cases (see Additional file 3).

Irrespective of the proportion of farms initially infected, their prevalence, and their centrality in the animal trade network, no spontaneous extinction was predicted at the metapopulation scale over the nine years of simulation. In particular, even in the case where only 1% of the farms were weakly initially infected (scenario A), the probability of persistence of the infection in the metapopulation was equal to 1.

**Figure 3:**
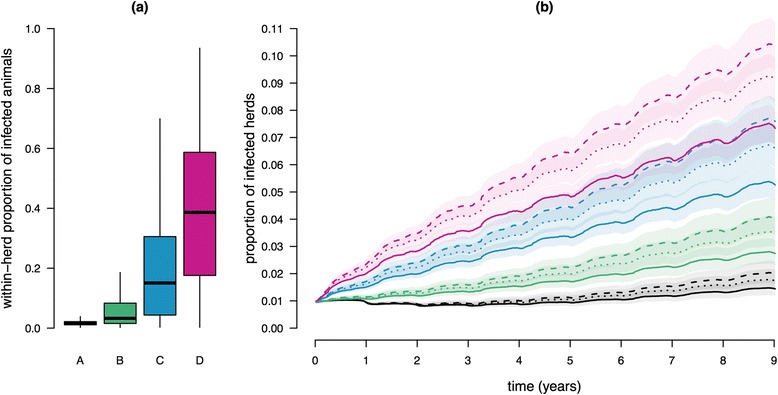
**Infection dynamics in the metapopulation of dairy cattle farms in Brittany - 1% of the farms initially infected.** (**A**) Distribution of the within-herd proportion of infected animals among initially infected farms for the four levels considered as initial conditions: very low (A, grey), low (B, green), medium (C, blue) and high (D, red). (**B**) Proportion of infected farms in the metapopulation over time (lines represent medians over 1000 runs for each scenario). Distinct colours correspond to different intra-herd levels of infection in initially infected farms (as in (**A**)). Line style corresponds to the type of sampling of initially infected farms: uniformly (solid line), proportional to the out-degree (dotted line), and proportional to the out-strength (dashed line). Coloured shaded areas represent empirical confidence cones (percentiles 0.10 and 0.90).

**Figure 4:**
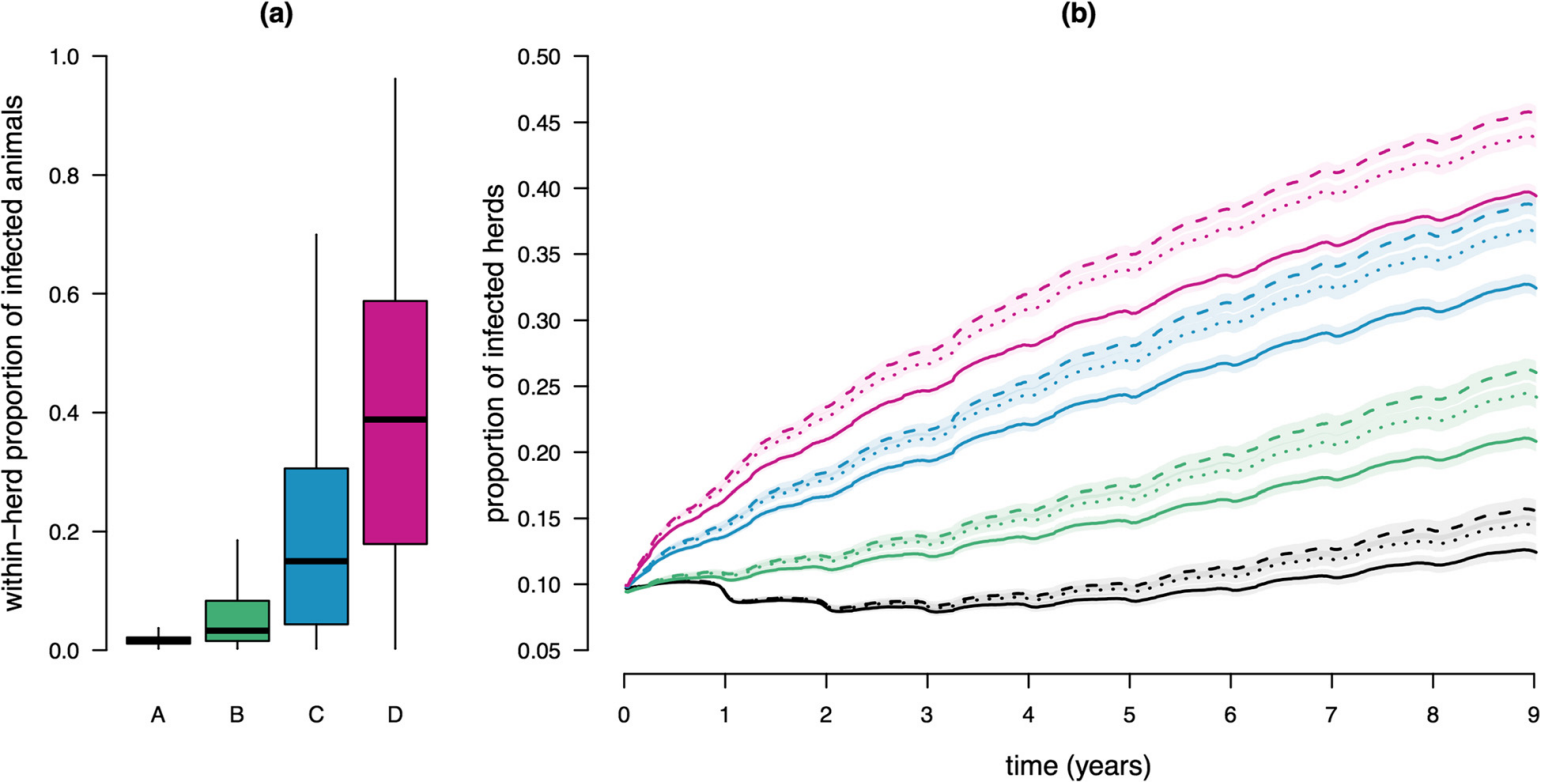
**Infection dynamics in the metapopulation of dairy cattle farms in Brittany - 10% of the farms initially infected.** (**A**) Distribution of the within-herd proportion of infected animals among initially infected farms for the four levels considered as initial conditions: very low (A, grey), low (B, green), medium (C, blue) and high (D, red). (**B**) Proportion of infected farms in the metapopulation over time (lines represent medians over 1000 runs for each scenario). Distinct colours correspond to different intra-herd levels of infection in initially infected farms (as in (**A**)). Line style corresponds to the type of sampling of initially infected farms: uniformly (solid line), proportional to the out-degree (dotted line), and proportional to the out-strength (dashed line). Coloured shaded areas represent empirical confidence cones (percentiles 0.10 and 0.90).

On the contrary, the speed and amplitude of *Map* spread between the farms of the metapopulation were largely affected by the proportion of initially infected farms and the level of infection in these farms (Figures 3 and 4). As expected, the larger was the proportion of farms initially infected and the greater their within-herd prevalence, the more numerous were the newly infected farms. In the case 1% of the farms were infected prior to *Map* propagation into the metapopulation and regardless of the way they were sampled, the number of incident farms was increased by 0.2% (ratio of 1.2) to 9% (ratio of 10) in 9 years, when increasing the level of within-herd prevalence (Figure 3). However, for a given sampling procedure of the initially infected farms, this relationship was not simply linear. Starting with 10% of the farms initially infected, the same increasing trend was observed but with much steeper slopes (e.g. the fraction of infected farms can increase from 10% to more than 40% in the worst case scenario, red lines in Figure 4). Regardless of the features of initial infection, the prevalence of infected farms at the regional level did not reach a steady-state but was still increasing after 9 years. Interestingly, when starting with 1% of the farms initially infected at the lowest level of within-herd prevalence (scenario A at lowest risk of *Map* regional spread and persistence, black curves in Figure 3), the number of infected farms decreased during 3 years prior to growing up. This is related to the occurrence of more local extinctions than of newly infected farms. In addition, the sampling scheme of initially infected farms also affected *Map* spread. Specifically, a selection of primarily infected farms favouring those with high out-degree or out-strength provided very similar results, and led to a faster spread and a larger number of infected farms than a uniform random selection.

**Figure 5:**
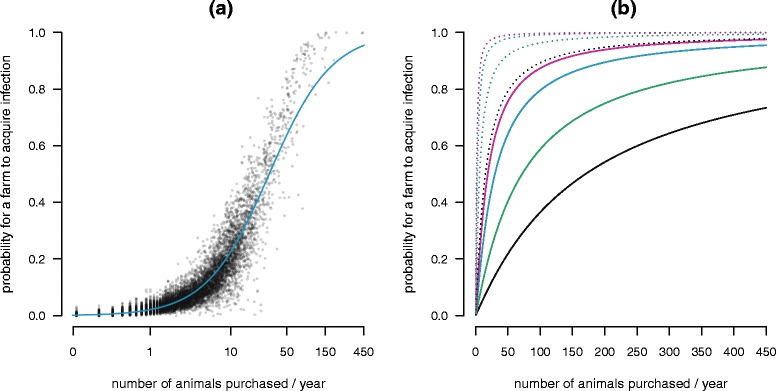
**Probability of acquiring infection at least once for Map-free farms as a function of the average number (over the nine years of data) of animals purchased per year.** (**A**) Each point corresponds to a farm in the metapopulation, which purchased at least one animal during the nine-year simulation. The scenario with 1% of the farms initially infected, uniformly sampled, and with medium levels of within-herd prevalence is shown. The solid blue line corresponds to the prediction of the general linear model with the logarithm of the number of purchased animals (in-strength) as explanatory variable. (**B**) Each curve corresponds to a different proportion of farms initially infected (1% - solid lines and 10% - dotted lines), and their levels of within-herd prevalence (very low - black, low - green, medium - blue and high - red). Only scenarios with initially infected farms uniformly sampled are shown. Lines correspond to the prediction of the general linear model with the logarithm of the number of purchased animals (in-strength) as explanatory variable.

We evidenced a large influence of farm characteristics on the probability of farm acquiring infection (Table 1), the best explanatory variable (based on both model selection criteria used) being the farm in-strength (number of animal purchased), especially after a logarithmic transformation (Figure 5A). Furthermore, the effect of the number of incoming animal movements on the probability of farm infection varied with the proportion of initially infected farms and their intra-herd prevalence of infection (Figure 5B). For high levels of initial infection, the probability of acquiring infection for *Map*-free farms was close to 1 if the number of animals introduced into the farm was larger than 25 animals per year. Also, starting from 10% of the farms initially infected at a medium level of within-herd prevalence, the probability of farm infection for an average of 4 animals purchased per year was higher than 0.5. At the opposite, when only 1% of the farms were initially weakly infected, the probability of acquiring infection for disease-free farms steadily increased with the number of animals purchased and never reached 1 in nine years of regional pathogen spread.

**Table 1.** **Results of the general linear regression for the probability of acquiring infection for** ***Map*** **-free farms**

**Explanatory variable included in the model**	**AIC** ^**a**^	**McFadden’s adjusted R** ^**2**^
Out-degree	815 747	0.026
Size	808 385	0.035
Out-strength	752 264	0.11
In-degree	544 260	0.37
Polarity	536 490	0.38
In-strength	306 029	0.67
All	212 280	0.79
Log(In-strength)	130 056	0.90

^a^The best model corresponds to the smallest AIC and the largest McFadden’s adjusted R^2^. All the *p*-values associated to variables in all models are < 0.05.

**Figure 6:**
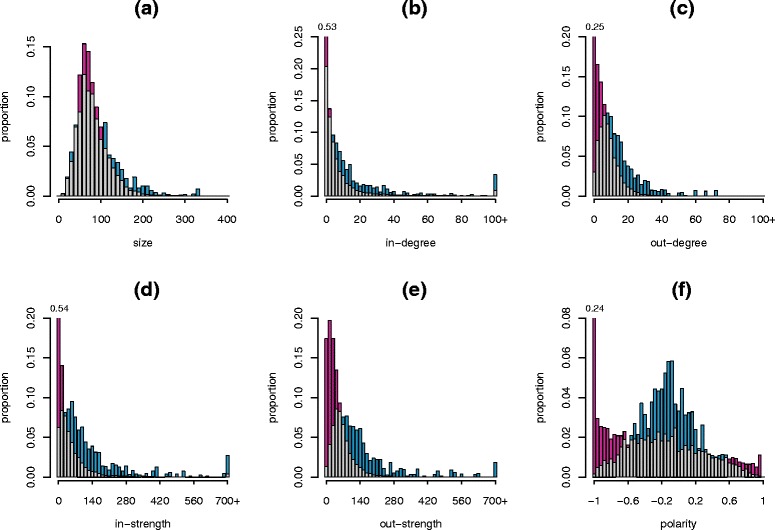
**Probability distributions of farms’ characteristics in the whole metapopulation (red) and among the secondarily infected farms that transmitted the disease to tertiary cases (blue).** Data used are aggregated over the whole period (2005-2013). Intersections between histograms in different populations are in grey. The scenario with 1% of the farms initially infected, uniformly sampled, and with medium levels of within-herd prevalence is shown (blue histograms). (**A**) Size; (**B**) In-degree (number of farms from which animals are purchased); (**C**) Out-degree (number of farms to which animals are sold); (**D**) In-strength (number of animals purchased); (**E**) Out-strength (number of animals sold); (**F**) Polarity (by construction, sellers have values <0, buyers have values >0).

The occurrence of new tertiary infections at the farm level caused by incident farms (secondary cases) was influenced by the characteristics of incident farms. Distributions of herd size, in and out-degree, in and out-strength, and polarity among incident farms generating tertiary cases were significantly different (*p* < 2.2e-16) from distributions of these same characteristics within the whole set of farms (Figure 6). Incident farms with herd size larger than 110 animals, with more than 8 outgoing connections and more than 70 animals sold, and with a polarity between −0.6 and 0.25 (rather seller behaviour) were more likely to transmit the disease. In particular for polarity, more than 50% of the infective incident farms behave like wholesalers. They correspond to farms with both a high risk to acquire infection and a high propensity to spread the pathogen when infected.

**Figure 7:**
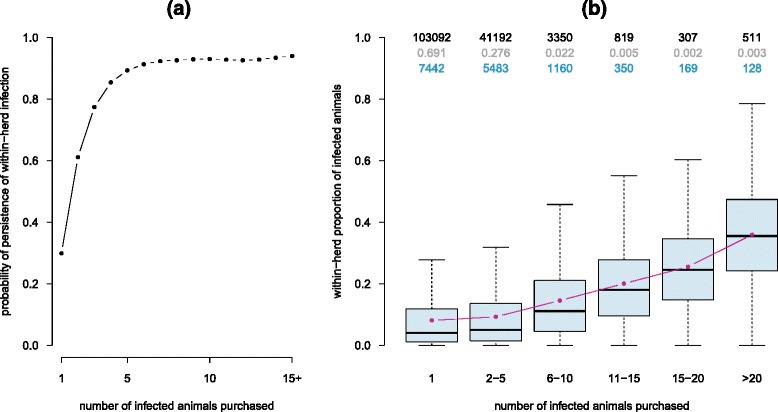
**Probability of persistence and within-herd proportion of infected animals in secondarily infected farms.** The scenario with 1% of the farms initially infected, uniformly sampled, and with medium levels of within-herd prevalence is shown. (**A**) Probability of persistence of within-farm infection 5 years after infection set up (corresponding to the introduction of the first animal) as a function of the number of infected animals purchased during the same period. (**B**) Within-herd prevalence at five years of infection duration according to the number of infected animals purchased by the farm over these five years. Each box contains values between the first and the third quartiles. Horizontal lines outside boxes correspond to the first quartile – 1.5x interquartile range and the third quartile + 1.5x interquartile range. Red dots correspond to mean values and thick horizontal lines to medians. For each range of the number of infected animals purchased, top values correspond to the number (black) and the proportion (grey) of incident farms in which infection was still persistent five years after the pathogen introduction over the whole set of runs. As a given farm can be counted several times, the number of distinct farms used to build the box plot is also provided (blue).

As expected, the probability of persistence of *Map* infection five years after the infection onset at the farm scale highly increased with the number of infected animals introduced during the infection duration (Figure 7A). A single *Map* introduction led to the same probability as observed in the case of an isolated farm. A second introduction of the pathogen was predicted to more than double the probability of persistence. For more than five *Map* introductions in nine years, the probability of persistence was around 90%. In addition, the within-farm infection burden also was influenced by the number of infected animals introduced during the period of infection (Figure 7B). The within-herd prevalence of infection five years after farm infection in farms introducing a single infected animal was very similar to the prevalence predicted in an isolated farm. In farms receiving more than one infected animal, the prevalence reached increased with the number of infected animals introduced. However, this effect was mitigated when increasing the severity of the initial state (with respect to the proportion of farms infected and their within-herd prevalence) prior to pathogen spread at the metapopulation scale (data not shown). No effect of other farm characteristics on the within-herd prevalence was shown.

**Figure 8:**
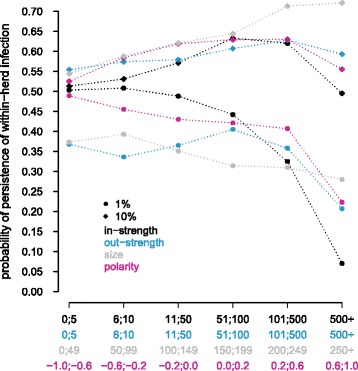
**Probability of persistence of the within-farm infection five years after infection set up as a function of farms’ characteristics.** Two scenarios are shown: 1% of the farms initially infected, uniformly sampled, and with medium levels of within-herd prevalence (circles) and 10% of the farms initially infected, uniformly sampled, and with high levels of within-herd prevalence (diamonds). The four farms’ characteristics tested are the number of animals purchased (in-strength: black), the number of animals sold (out-strength: blue), the polarity (red), and the size (grey). Each of these characteristics is divided into six intervals (min and max values for each interval are indicated), and the average probability of persistence is calculated for each group.

More unexpectedly, for 1% of initially infected farms at moderate levels, the probability of persistence at the farm scale decreased when the in-strength increased, whereas this impact was less pronounced for the other characteristics, especially for herd size and out-strength (Figure 8). Farms with a large number of incoming animal movements, and therefore with a high probability of being infected, showed a very low risk of persistent infection. This trend was not present for a scenario starting with 10% of initially infected farms with high levels of within-farm infection. For this scenario, the probability of persistence of within-farm infections either increased when size or network-related characteristics of farms increased, or stood relatively stable with respect to these characteristics.

## Discussion

We presented here the first multi-scale spatio-temporal model to predict *Map* spread in a metapopulation of dairy cattle farms. This model couples within-farm dynamics through observed between-farm animal trade movements. Compared to the few published models of *Map* spread at a regional scale [32,33], this model is original as it simultaneously accounts for stochastic demographic and infection dynamics within dairy farms, indirect local transmission, and data on animal trade, herd size, and farm management. This level of detail is required to accurately represent *Map* spread. Indeed, paratuberculosis is a slow progressive disease with chronic infection and whose prevalence of infection is expected to largely vary among infected farms as well as over the course of infection in a given farm [31]. Due to the fidelity of the model in representing mechanisms governing *Map* spread, the intensive simulations performed in this study as well as their analysis help to provide a better understanding of the within and between-farm infection dynamics.

Cattle movements are modelled explicitly based on real trade data, which allows us for accounting for the time-varying nature of such a network. In Europe, most national cattle identification databases are well documented, movements being recorded daily. The analysis of the FCID showed that connections between nodes vary among years, a very small proportion of the links being preserved over time [5]. Moreover, the number of farms decreases due to farm merging. The available data also allows us to reproduce the demographic trends over the considered period, specifically for each farm. Indeed, herd size and farm management (especially the renewal of adults, culling, and trade) can be highly variable among years. Our model is data-driven, which can be viewed as a strength or as a limitation. On the one hand, this enables us to provide a realistic representation of interacting farms at a regional scale, and therefore to more precisely identify the mechanisms involved in the spread of pathogens and the main drivers for their subsequent control. On the other hand, it constrains the simulation period to the range of observed data. This limitation could be overcome if it was possible to generate network dynamics for unobserved time horizons. Therefore, there is an urgent need for predictive models of animal trade movements in order to not only represent past and current situations, but also carry on long-term predictions. The availability of predicted animal trade movements, incorporated into regional models of pathogen spread, would also provide a valuable hand in assessing control measures based on movement regulations, for example by accounting for farm epidemiological statuses, one of the major options to prevent *Map* introduction in *Map*-free farms.

The model is based on two main simplifying assumptions. First, we assumed that farmers having both dairy and beef production units or having a fattening activity manage the animals related to different activities in separate buildings. Therefore, *Map* transmission between units is expected to be low and negligible. Consequently, we accounted only for dairy and cross-bred females in the model. Movements of beef animals and of males were not represented. Second, as no data was available at the time of the study on *Map* prevalence (proportion of infected farms or infected animals), we assumed that the risk of purchasing an infected animal from outside the metapopulation considered was the same as the risk within the metapopulation. In the absence of control measures in or outside the modelled area, assuming such a homogeneous risk is relevant. However, such an assumption should be relaxed to account for a spatial heterogeneity in *Map* prevalence, especially if control measures implemented in the region considered and outside this region are not the same.

According to our model predictions, *Map* infection is highly persistent over time at a regional scale, regardless of the initial prevalence of infection. The number of new infections of farms is sufficient to avoid local extinctions. Hence, even for regions with a low proportion of infected farms, *Map* spread will not fade out spontaneously, without the use of effective control strategies. This is consistent with the observed situation in the considered region, Brittany (in Northwestern France), characterized by a high density of dairy cattle, where bovine paratuberculosis is known as endemic [39]. Similar patterns are observed in a large number of other regions in the world [15]. Theoretical work carried out on the persistence of infectious diseases in a metapopulation mainly concerned curable diseases. In those contexts, the probability of extinction of the infection has been shown to be highly related to the rate of animal movements [40,41]. For chronic diseases such as paratuberculosis, extinction will not occur at a regional scale without human interventions, and this even for low movement rates among populations.

Our model predictions support very high proportions of infected farms, showing a continuous increase in the number of infected farms over a nine-year period, irrespective of the proportion of initially infected farms and their intra-herd prevalence of infection. This is in agreement with current knowledge, the prevalence of *Map* infection being assumed to be higher than 50% and still increasing in most countries with a significant dairy industry [14]. The screening of bovine paratuberculosis in the field is rendered very difficult due to the long incubation period and to the low sensitivity of available diagnostic tests currently used in routine [42]. Therefore, the true prevalence of infection remains mostly unknown. Our model provides valuable indications on *Map* spread at a regional scale and its possible drivers. We can expect that, without any control measures, *Map* infection will spread to all reachable farms, i.e. all those purchasing animals even occasionally. The probability of being infected at least once during a period is related to the number of animals purchased over that period. Considering the most probable levels of infection in a region with a high density of dairy farms [14,16], we can derive from our model predictions that farms buying a minimum of 3 animals per year have a risk of acquiring infection during a period of 9 years greater than 0.5.

Incoming and outgoing movements to and from a farm localized in a metapopulation are expected to modify pathogen spread in that farm compared to pathogen spread in an isolated farm. As expected, reintroducing *Map* infected animals in infected farms led to a faster spread and a greater persistence at the farm level. Moreover, the probability of *Map* reintroduction increased with the number of incoming movements. However, infection persistence may decrease with an increase in the number of animals exchanged. This clearly evidences the interaction between population dynamics and infection dynamics. At low prevalence of infection in the metapopulation, the risk of purchasing infected animals is low. A high turnover (to keep constant the herd size) within farms associated with a large purchasing rate increase the probability of removing infected animals and therefore decrease persistence. On the contrary, when the prevalence is high, persistence is no longer affected by the within-herd turnover. The worst situation then consists in farms with a high number of incoming movements but a low turnover. This can occur for farms that enlarge their livestock through purchases, an increasingly widespread behaviour.

While the drivers of *Map* spread at a regional scale are not expected to vary with its speed, the simulated propagation is probably much faster than the one in the field at the emergence of paratuberculosis in Western France. Indeed, much fewer trade exchanges occurred between farms during the last century, whereas farms were more numerous [37]. The increase in animal trade movements and farm merging could have led to a significant increase in *Map* spread. However, accurate data on animal trade corresponding to the early stages of *Map* invasion would be necessary to validate these hypotheses.

Our model has enabled a better understanding of *Map* spread at a regional scale, as related to herd population dynamics and time-varying trade patterns between farms. This model can be used to predict *Map* spread in any dairy farming region, as long as data on herd demography, farm management, and animal movements is available. In the absence of current knowledge on the exact epidemiological situation in the field, this model is a valuable tool for evaluating and prioritizing combined control measures for various within-herd and regional levels of infection.

## Additional files

Additional file 1:
**The within-herd model of Map spread.** This file contains equations of the within-herd dynamics and definition and values of parameters. Additional file 2:
**Network characteristics.** This file contains graphs representing distributions of herd size and of characteristics of the network describing cattle trade data for Brittany over the period 2005-2013. Additional file 3:
**Population dynamics calibration.** This file contains graphs on population dynamics (data versus simulation).
