# Supplementary material for: Modelling of paratuberculosis spread between dairy cattle farms at a regional scale
Source: Vet Res. 2015 Sep 25;46:111. doi: 10.1186/s13567-015-0247-3 (PMC4583165; doi:10.1186/s13567-015-0247-3)
Supplement: Additional file 1: — The within-herd model of Map spread. This file contains equations of the within-herd dynamics and definition and values of parameters. [file 13567_2015_247_MOESM1_ESM.pdf]

Additional file 1

The within-herd model of Map spread

A Equations for the within-herd dynamic

The description of the within-herd model we used corresponds to an update of the model described in [31], which we formalized here for the first time. The modifications we introduced are mainly related to flows corresponding to births and exits, in order to take into account population dynamics specifically for each herds.
Specifically, births are plugged into the model based on data and male calves are no longer considered. In addition, the density dependence of exit rates has been removed. The population dynamics is now stabilized through specific exit rates for each herd, the purchase and sale of animals being defined by the data on cattle movements. Information in the database used (FCID, see section entitled "Animal trade data and network representation" in the main manuscript) does not allow us to calculate mortality and culling rates per parity (it is not possible to obtain the number of calvings per animal from the information available). Therefore, mortality and culling rates are calculated by age group for adults (1, 2, 3, 4 and 5+ correspond to the number of years following the entry into the adult stage).
The set of parameters used are described in Additional files 2 and 3.

Notations

In the following equations, $X_{(t,a)}$ is the number of animals in health state X at time t and age a. Age is given in weeks until the entry in the adult stage (from 1 to ad, with intermediary stages w for weaning age, y for young heifers age, h for heifers age and u for maximal age in the susceptible compartments), then by age group (A_1 to A_{5+}). Some variables can have a prefix: "b" for births in health states X (bX), "N" for animals transiting between two health states ($\mathrm{N}X$) and "s" for exits (mortality and culling) (sX). After entering the adult stage, flows corresponding to aging are noted using a superscript ng, whereas those remaining in the same age group are noted using a superscript sg. $N_{(t,a)}$ is the number of animals of age a at time t. Average duration in health states are noted by v_X. The remaining terms used are defined when introduced.

Equations for the updating of variables describing health states

In this section we introduce the equations for the updating of variables corresponding to the health states, for a given herd i. Connexions between herds are possible by adding a term representing animal movements in the equations below.

Susceptible (S) and Resistant (R)

$$S_{(t+1,a=1)} = \ bS - \mathrm{N}T_{(t+1,a)}$$

$$S_{(t+1,a\in[2;52])} = \left[S_{(t,a-1)} - sS_{(t,a-1)}\right] - \mathrm{N}T_{(t+1,a)}$$

$$R_{(t+1,a=53)} = S_{(t,a-1)} - sS_{(t,a-1)}$$

$$R_{(t+1,a\in[54;ad])} = R_{(t,a-1)} - sR_{(t,a-1)}$$

$-$

$$R_{(t+1,a=A_1)} = R^{sg}_{(t,A_1)} - sR^{sg}_{(t,A_1)} + R_{(t,ad)} - sR_{(t,ad)}$$

$$R_{(t+1,A_i\in[A_2;A_4])} = R^{sg}_{(t,A_i)} - sR^{sg}_{(t,A_i)} + R^{ng}_{(t,A_{i-1})} - sR^{ng}_{(t,A_{i-1})}$$

$$R_{(t+1,a=A_{5+})} = R_{(t,A_i)} - sR_{(t,A_i)} + R^{ng}_{(t,A_{i-1})} - sR^{ng}_{(t,A_{i-1})}$$

Transiently infected (T)

$$T_{(t+1,a=1)} = \ bT + \mathrm{N}T_{(t+1,a)}$$

$$T_{(t+1,a\in[2;52])} = \left[T_{(t,a-1)} - sT_{(t,a-1)}\right] - \mathrm{N}L_{(t+1,a)} + \mathrm{N}T_{(t+1,a)}$$

$$T_{(t+1,a\in[53;ad])} = \left[T_{(t,a-1)} - sT_{(t,a-1)}\right] - \mathrm{N}L_{(t+1,a)}$$

Latently infected (L)

$$L_{(t+1,a=2)} = \ \mathrm{N}L_{(t+1,a)}$$

$$L_{(t+1,a\in[3;h])} = \ \left[L_{(t,a-1)} - sL_{(t,a-1)}\right] + \mathrm{N}L_{(t+1,a)}$$

$$L_{(t+1,a\in[h+1;ad])} = \ \left[L_{(t,a-1)} - sL_{(t,a-1)}\right] - \mathrm{N}Is_{(t+1,a)} + \mathrm{N}L_{(t+1,a)}$$

$-$

$$L_{(t+1,A_1)} = \ \left[L^{sg}_{(t,A_1)} - sL^{sg}_{(t,A_1)} + L_{(t,ad)} - sL_{(t,ad)} + T_{(t,ad)} - sT_{(t,ad)}\right] - \mathrm{N}Is_{(t+1,A_1)}$$

$$L_{(t+1,A_i\in[A_2;A_4])} = \left[L^{sg}_{(t,A_i)} - sL^{sg}_{(t,A_i)} + L^{ng}_{(t,A_{i-1})} - sL^{ng}_{(t,A_{i-1})}\right] - \mathrm{N}Is_{(t+1,A_i)}$$

$$L_{(t+1,A_{5+})} = \left[L_{(t,A_{5+})} - sL_{(t,A_{5+})} + L^{ng}_{(t,A_4)} - sL^{ng}_{(t,A_4)}\right] - \mathrm{N}Is_{(t+1,A_{5+})}$$

Subclinically infected (Is)

$$Is_{(t+1,a=h+1)} = \ \mathrm{N}Is_{(t+1,a)}$$

$$Is_{(t+1,a\in[h+2;ad])} = \ \left[Is_{(t,a-1)} - sIs_{(t,a-1)}\right] - \mathrm{N}Ic_{(t+1,a)} + \mathrm{N}Is_{(t+1,a)}$$

$-$

$$Is_{(t+1,A_1)} = \ \left[Is^{sg}_{(t,A_1)} - sIs^{sg}_{(t,A_1)} + Is_{(t,ad)} - sIs_{(t,ad)}\right] - \mathrm{N}Ic_{(t+1,A_1)} + \mathrm{N}Is_{(t+1,A_1)}$$

$$Is_{(t+1,A_i\in[A_2;A_4])} = \left[Is^{sg}_{(t,A_i)} - sIs^{sg}_{(t,A_i)} + Is^{ng}_{(t,A_{i-1})} - sIs^{ng}_{(t,A_{i-1})}\right] - \mathrm{N}Ic_{(t+1,A_i)} + \mathrm{N}Is_{(t+1,A_i)}$$

$$Is_{(t+1,A_{5+})} = \left[Is_{(t,A_{5+})} - sIs_{(t,A_{5+})} + Is^{ng}_{(t,A_4)} - sIs^{ng}_{(t,A_4)}\right] - \mathrm{N}Ic_{(t+1,A_{5+})} + \mathrm{N}Is_{(t+1,A_{5+})}$$

Clinically infected (Ic)

$$Ic_{(t+1,a=h+2)} = \text{N}Ic_{(t+1,a)}$$

$$Ic_{(t+1,a\in[h+3;ad])} = \left[Ic_{(t,a-1)} - sIc_{(t,a-1)}\right] + \text{N}Ic_{(t+1,a)}$$

$-$

$$Ic_{(t+1,A_1)} = \left[Ic^{sg}_{(t,A_1)} - sIc^{sg}_{(t,A_1)} + Ic_{(t,ad)} - sIc_{(t,ad)}\right] + \text{N}Ic_{(t+1,A_1)}$$

$$Ic_{(t+1,A_i\in[A_2;A_4])} = \left[Ic^{sg}_{(t,A_i)} - sIc^{sg}_{(t,A_i)} + Ic^{ng}_{(t,A_{i-1})} - sIc^{ng}_{(t,A_{i-1})}\right] + \text{N}Ic_{(t+1,A_i)}$$

$$Ic_{(t+1,A_{5+})} = \left[Ic_{(t,A_{5+})} - sIc_{(t,A_{5+})} + Ic^{ng}_{(t,A_4)} - sIc^{ng}_{(t,A_4)}\right] + \text{N}Ic_{(t+1,A_{5+})}$$

Equations describing flows

Births (bX)

At each time step t, births are plugged into the model from data. This corresponds to the total number of births of dairy females, and is noted by $Births(t)$.

These births are then distributed into S and T states

$$bS_{(t)} = bS^R_{(t)} + bS^L_{(t)} + bS^{Is}_{(t)} + bS^{Ic}_{(t)} \quad \text{and} \quad bT_{(t)} = bT^L_{(t)} + bT^{Is}_{(t)} + bT^{Ic}_{(t)},$$

where

$$bS^R_{(t)} = nbC^R_{(t)}$$
$$bS^L_{(t)} = nbC^L_{(t)} - bT^L_{(t)}, \quad bT^L_{(t)} \sim Bin\left(nbC^L_{(t)}; p_L\right)$$
$$bS^{Is}_{(t)} = nbC^{Is}_{(t)} - bT^{Is}_{(t)}, \quad bT^{Is}_{(t)} \sim Bin\left(nbC^{Is}_{(t)}; p_{Is}\right)$$
$$bS^{Ic}_{(t)} = nbC^{Ic}_{(t)} - bT^{Ic}_{(t)}, \quad bT^{Ic}_{(t)} \sim Bin\left(nbC^{Ic}_{(t)}; p_{Ic}\right)$$

In equations above, $nbC^X_{(t)}$ is the number of female calves alive born at time t. It is obtained from $nbV^X_{(t)}$, the number of female calves born at time t, from cows in the health state X:

$$nbC^X_{(t)} \sim Bin\left(nbV^X_{(t)}; 1-\sigma_B\right), \text{where}$$

$$nbV^X_{(t)} = nbV^X_{(t,ad)} + \sum_{i=1}^{i=5} nbV^X_{(t,A_i)}, \text{and}$$

$$nbV^X_{(t,a)} \sim Multinomial\left(nbV_{(t,a)}, p_{(t,a)}\right), \text{ where}$$
$$p_{(t,a)} = \left(\frac{R_{(t,a)}}{N_{(t,a)}}, \frac{L_{(t,a)}}{N_{(t,a)}}, \frac{Is_{(t,a)}}{N_{(t,a)}}, \frac{Ic_{(t,a)}}{N_{(t,a)}}\right) \text{ with } N_{(t,a)} = \sum_{X\in(R,L,Is,Ic)} X_{(t,a)}, \text{ for } a \in \{ad, A_{i\in\{1,2,3,4,5\}}\}.$$

Irrespective to the health state, the number of births per age group, $nbV_{(t,a)}$, is given by:

$$nbV_{(t,a)} \sim Multinomial\left(Births(t), p_{(t,a)}\right), \text{ where}$$
$$p_{(t,a)} = \left(\frac{K_{(t,ad)}}{K_{(t)}}, \frac{K_{(t,A_1)}}{K_{(t)}}, \frac{K_{(t,A_2)}}{K_{(t)}}, \frac{K_{(t,A_3)}}{K_{(t)}}, \frac{K_{(t,A_4)}}{K_{(t)}}, \frac{K_{(t,A_5)}}{K_{(t)}}\right) \text{ with } K_{(t)} = \sum_{a\in\{ad,A_{i\in\{1,2,3,4,5\}}\}} K_{t,a}$$

and

$$K_{(t,ad)} = min\left(\sum_{i=1}^{i=5} N_{(t,A_i)} \times \sigma_{A(i)}; N_{(t,ad)}\right),$$
$$K_{(t,A_i)} = N_{(t,A_i)} \times \left(1 - \sigma_{A(i)}\right).$$

We note that at the age of moving in the adult group (ad), all the animals in the health state T enter the health state L ($L_{(t,ad)} = L_{(t,ad)} + T_{(t,ad)}$).

Change in age group (X^x)

$$X_{(t,A_i)} = X^{sg}_{(t,A_i)} + X^{ng}_{(t,A_i)}$$
$$X^{ng}_{(t,A_i)} \sim Bin\left(X_{(t,A_i)}; \frac{1}{\tau_{aa}}\right), \quad \text{where } X = \{R, L, Is, Ic\},$$

and τ_{aa} is the average time spent in each of age group A_1 to A_4.

Exits (sX)

The mortality of calves during the first week of life is applied at birth and defined above in the section concerning births.
From age 1 to ad, mortality and culling rates σ_x are defined as:

- $a \in \{1; 2\} \to \sigma_x = \sigma_{c1}$
- $a \in \{3; w\} \to \sigma_x = \sigma_{c2}$
- $a \in \{w + 1; ad\} \to \sigma_x = \sigma_{c3}$

Then, exits following death and culling write as:

for $a \in \{1; ad\}$:

$$sX_{(t,a)} \sim Bin\left(X_{(t,a)}; \sigma_x\right), \quad \text{where } X = \{S, R, T, L, Is, Ic\},$$

and for $a \in \{A_1;A_{5+}\}$:

$$sX^x_{(t,A_i)} \sim Bin\left(X^x_{(t,A_i)}; \sigma_{Ai}\right), \quad \text{where } X = \{R, L, Is\},$$
$$sIc^x_{(t,A_i)} \sim Bin\left(Ic^x_{(t,A_i)}; \frac{1}{v_{Ic}}\right),$$ where v_{Ic} is the mean time spent in the health state Ic.

New infections (S → T, except for *in-utero* transmission)

We have:

$$\text{N}T_{(t+1,a)} = inf^{c}_{(t+1,a)} + inf^{m}_{(t+1,a)} + inf^{l}_{(t+1,a)} + inf^{g}_{(t+1,a)}$$

Superscripts correspond to different possible routes of transmission, respectively colostrum (c), milk (m), local environment (l) and global environment (g). New infections by *in-utero* transmission are accounted for through births.

By age, the possible routes of infection are:

$$0 \underbrace{\longrightarrow}_{cmlg} 1 \underbrace{\rightarrow \cdots \rightarrow}_{mlg} \text{weaning} \underbrace{\rightarrow \cdots \rightarrow}_{lg} \text{grazing allowed} \underbrace{\rightarrow \cdots \rightarrow}_{\substack{\text{outdoor: } l \\ \text{indoor: } lg}} \text{limit of susceptibility}$$

Transmission through colostrum

It is considered that calves drink colostrum from their mothers during the first three days before drinking milk:

$$inf^{c}_{(t+1,a=1)} = \sum_{1}^{bS^{Is}_{(t)}} \left[Bern\left(1 - exp\left(-\frac{\beta_l \; q^{Is}_c}{\alpha}\right)\right)\right] + \sum_{1}^{bS^{Ic}_{(t)}} \left[Bern\left(1 - exp\left(-\frac{\beta_l \; q^{Ic}_c}{\alpha}\right)\right)\right],$$

with

$$q^{Is}_c \sim Bern(sh_{Is}) \times \left[3 \times b\left(10^5.Beta(8;8) + 1 + 10^3.Beta(1;25)\right)\right],$$
$$q^{Ic}_c \sim Bern(sh_{Ic}) \times \left[3 \times b\left(10^5.Beta(8;8) + 10^{(3+10.Beta(50;200))}\right)\right].$$

Transmission through milk

Regarding the age, we have:

$$a = 1: \quad inf^{m}_{(t+1,a)} \sim Bin\left(bS; 1 - exp\left(-\frac{\beta_l \; q_l \; \frac{4}{7}}{\alpha}\right)\right),$$
$$a \in \{2; w\}: \quad inf^{m}_{(t+1,a)} \sim Bin\left(\left[S_{(t,a-1)} - sS_{(t,a-1)}\right]; \left[1 - exp\left(-e^{(-\gamma(a-1))}.\frac{\beta_l \; q_l}{\alpha}\right)\right]\right),$$

with

$$q_l = \frac{7 \times d \times \left(Q^{milk}_{Is(t)} + Q^{milk}_{Ic(t)}\right)}{MilkTot_{(t)}},$$

where

$$Q^{milk}_{Is(t)} = 7 \times \varepsilon \times g_{Is} \times \left(\sum_{1}^{nbExcr^{Is}_{(t)}} (10^5.Beta(8;8)) + \sum_{1}^{nbLac^{Is}_{(t)}} (1 + 10^3.Beta(1;25)) \right),$$

$$Q^{milk}_{Ic(t)} = 7 \times \varepsilon \times g_{Ic} \times \left(\sum_{1}^{nbExcr^{Ic}_{(t)}} (10^5.Beta(8;8)) + \sum_{1}^{nbLac^{Ic}_{(t)}} 10^{(3+10.Beta(50;200))} \right),$$

$$MilkTot_{(t)} = 7 \times \varepsilon \times \left(nbLac^{R}_{(t)} + g_L.nbLac^{L}_{(t)} + g_{Is}.nbLac^{Is}_{(t)} + g_{Ic}.nbLac^{Ic}_{(t)} \right).$$

with

$$nbExcr^{Is}_{(t)} \sim Bin\left(nbLac^{Is}_{(t)}; sh_{Is}\right),$$

$$nbExcr^{Ic}_{(t)} \sim Bin\left(nbLac^{Ic}_{(t)}; sh_{Ic}\right),$$

$$nbLac^{R}_{(t)} \sim Bin\left(\sum_{i=1}^{i=5} R_{(t,A_i)}, prop\right),$$

$$nbLac^{L}_{(t)} \sim Bin\left(\sum_{i=1}^{i=5} L_{(t,A_i)}, prop\right),$$

$$nbLac^{Is}_{(t)} \sim Bin\left(\sum_{i=1}^{i=5} Is_{(t,A_i)}, prop\right),$$

$$nbLac^{Ic}_{(t)} \sim Bin\left(\sum_{i=1}^{i=5} Ic_{(t,A_i)}, prop\right).$$

Local transmission (in collective pens, $a \in [1;u]$)

$$inf^{l}_{(t+1,a)} \sim Bin\left(S_{(t,a-1)} - sS_{(t,a-1)}; p^{i}_{inf(t+1,a)}\right),$$

where

during housing period:

$$p^{i}_{inf(t+1,a)} = 1 - exp\left(-e^{(-\gamma(a-1))}.\frac{\beta_c E^{i}_{(t+1)}}{\alpha N^{i}_{(t+1)}}\right), \text{ and}$$

during grazing period, regarding the age,

$$a \in [1;26]: \quad p^{i}_{inf(t+1,a)} = 1 - exp\left(-e^{(-\gamma(a-1))}.\frac{\beta_c E^{i}_{(t+1)}}{\alpha N^{i}_{(t+1)}}\right),$$

$$a \in [27;u]: \quad p^{i}_{inf(t+1,a)} = 1 - exp\left(-e^{(-\gamma(a-1))}.\frac{\beta_o E^{i}_{(t+1)}}{\alpha N^{i}_{(t+1)}}\right).$$

$N^{i}_{(t+1)}$ is the total number of animals in environment i, across all health states, and E^{i} represents the quantity of bacteria in the environment, with i corresponding to the specific area.

Global transmission

This occurs up to the age allowing to go to the pasture (26 weeks - 6 months) during the grazing period, and up to the age limit for sensitivity (u) during the housing period:

$$inf^{g}_{(t+1,a)} \sim Bin\left(S_{(t,a)} - sS_{(t,a)}; p^{g}_{inf(t+1,a)}\right),$$

where

$$p^{g}_{inf(t+1,a)} = 1 - exp\left(-exp[-h(a-1)].\frac{\beta_g E^{g}_{(t+1)}}{\alpha N^{g}_{(t+1)}}\right), \quad \text{with} \quad E^{g}_{(t+1)} = \sum_{i=1}^{i=5} E^{\text{INT}i}_{(t+1)}$$

$E^{\text{INT}i}$ represents the quantity of bacteria in the environment where $\text{INT}i$ corresponds to a specific area (see the section below about the dynamics of bacteria in the environments).

New latently infected (T → L)

For $a < ad$:

$$\text{N}L_{(t+1,a\in[2;ad-1])} \sim Bin\left(T_{(t,a)} - sT_{(t,a)}; \frac{1}{v_T}\right).$$

After age ad, there are no more animals in T state.

New subclinically infected (L → Is)

For heifers:

$$\text{N}Is_{(t+1,a\in[h+1;ad])} \sim Bin\left(L_{(t,a)} - sL_{(t,a)}; \frac{1}{v_L}\right).$$

For cows:

$$\text{N}Is_{(t+1,A_i)} \sim Bin\left(n; \frac{1}{v_L}\right),$$

with, regarding the age,

$$A_1 \to n = \left[L^{sg}_{(t,A_1)} - sL^{sg}_{(t,A_1)} + L_{(t,ad)} - sL_{(t,ad)} + T_{(t,ad)} - sT_{(t,ad)}\right],$$
$$\{A_2; A_4\} \to n = \left[L^{sg}_{(t,A_i)} - sL^{sg}_{(t,A_i)} + L^{ng}_{(t,A_{i-1})} - sL^{ng}_{(t,A_{i-1})}\right],$$
$$A_{5+} \to n = \left[L_{(t,A_{5+})} - sL_{(t,A_{5+})} + L^{ng}_{(t,A_4)} - sL^{ng}_{(t,A_4)}\right].$$

New clinically infected (Is → Ic)

For heifers:

$$\text{N}Ic_{(t+1,a\in[h+1;ad])} \sim Bin\left(Is_{(t,a)} - sIs_{(t,a)}; \frac{1}{v_{Is}}\right).$$

For cows:

$$\text{N}Ic_{(t+1,A_i)} \sim Bin\left(n; \frac{1}{v_{Is}}\right),$$

with

$$\begin{aligned}
A_1 &\rightarrow n = \left[Is^{sg}_{(t,A_1)} - sIs^{sg}_{(t,A_1)} + Is_{(t,ad)} - sIs_{(t,ad)}\right], \\
\{A_2; A_4\} &\rightarrow n = \left[Is^{sg}_{(t,A_i)} - sIs^{sg}_{(t,A_i)} + Is^{ng}_{(t,A_{i-1})} - sIs^{ng}_{(t,A_{i-1})}\right], \\
A_{5+} &\rightarrow n = \left[Is_{(t,A_{5+})} - sIs_{(t,A_{5+})} + Is^{ng}_{(t,A_4)} - sIs^{ng}_{(t,A_4)}\right].
\end{aligned}$$

New resistant (S → R)

At age u, the transition from compartment S to compartment R is done in a deterministic way.

Dynamics of bacteria in the environments (E)

The composition of the environments according to the season is the following:

$$\text{Grazing} : a = \overbrace{1 \cdots\cdots w}^{\text{INT1}}\overbrace{\cdots\cdots 26}^{\text{INT2}}\overbrace{\cdots\cdots\cdots\cdots h}^{\text{EXT1+EXT2}}\overbrace{\cdots\cdots\cdots ad}^{\text{EXT3}}$$

$$\text{Housing} : a = \overbrace{1 \cdots\cdots w}^{\text{INT1}}\overbrace{\cdots\cdots\cdots\cdots y}^{\text{INT2}}\overbrace{\cdots\cdots\cdots h}^{\text{INT3}}\overbrace{\cdots\cdots\cdots ad}^{\text{INT4}}$$

Dynamics of bacteria in the environments (E) are defined below:

$E^{\text{INT1}}_{(t+1)} = E^{\text{INT1}}_{(t)}.(1 - \mu^{int}_g) + Q^{\text{TNS}}_{(t)}$. If pens are empty, it becomes $E^{\text{INT1}}_{(t+1)} = E^{\text{INT1}}_{(t+1)}.(1 - \mu_{cp})$, where Q represents the quantity of bacteria shed.

During grazing period, we have:

$$E^{\text{INT2}}_{(t+1)} = E^{\text{INT2}}_{(t)}.(1-\mu_g^{int}) + Q^{\text{Ts1}}_{(t)}$$

$$If \quad \sum_{a=w+1}^{a=26} SR_{t,a} + \sum_{a=w+1}^{a=26} T_{t,a} + \sum_{a=w+1}^{a=26} L_{t,a} == 0 \quad then \quad E^{\text{INT2}}_{(t+1)} = E^{\text{INT2}}_{(t+1)}.(1-\mu_{cp})$$

$$E^{\text{INT3}}_{(t+1)} = E^{\text{INT3}}_{(t)}.(1-\mu_g^{int})$$

$$E^{\text{INT4}}_{(t+1)} = E^{\text{INT4}}_{(t)}.(1-\mu_g^{int})$$

$$E^{\text{INT5}}_{(t+1)} = E^{\text{INT5}}_{(t)}.(1-\mu_g^{int})$$

$$E^{\text{EXT1}}_{(t+1)} = E^{\text{EXT1}}_{(t)}.(1-\mu_g^{ext}) + Q^{\text{Ts2}}_{(t)}$$

$$E^{\text{EXT2}}_{(t+1)} = E^{\text{EXT2}}_{(t)}.(1-\mu_g^{ext}) + Q^{\text{Ty}}_{(t)}$$

$$E^{\text{EXT3}}_{(t+1)} = E^{\text{EXT3}}_{(t)}.(1-\mu_g^{ext}) + Q^{\text{Th}}_{(t)} + Q^{\text{Ish}}_{(t)} + Q^{\text{Ich}}_{(t)}.$$

During housing period, we have:

$$E^{\text{INT2}}_{(t+1)} = E^{\text{INT2}}_{(t)}.(1-\mu_g^{int}) + Q^{\text{Ts1}}_{(t)} + Q^{\text{Ts2}}_{(t)}$$

$$If \quad \sum_{a=w+1}^{a=52} SR_{t,a} + \sum_{a=w+1}^{a=52} T_{t,a} + \sum_{a=w+1}^{a=52} L_{t,a} == 0 \quad then \quad E^{\text{INT2}}_{(t+1)} = E^{\text{INT2}}_{(t+1)}.(1-\mu_{cp})$$

$$E^{\text{INT3}}_{(t+1)} = E^{\text{INT3}}_{(t)}.(1-\mu_g^{int}) + Q^{\text{Ty}}_{(t)}$$

$$E^{\text{INT4}}_{(t+1)} = E^{\text{INT4}}_{(t)}.(1-\mu_g^{int}) + Q^{\text{Th}}_{(t)} + Q^{\text{Ish}}_{(t)} + Q^{\text{Ich}}_{(t)}$$

$$E^{\text{INT5}}_{(t+1)} = E^{\text{INT5}}_{(t)}.(1-\mu_g^{int}) + Q^{\text{Is}}_{(t)} + Q^{\text{Ic}}_{(t)}$$

$$E^{\text{EXT1}}_{(t+1)} = 0, \quad E^{\text{EXT2}}_{(t+1)} = 0, \quad E^{\text{EXT3}}_{(t+1)} = 0.$$

Shed quantities of bacteria are defined, regarding the health states and the age, by:

unweaned calves T:

$$Q^{\text{Tns}}_{(t)} = \sum_{a=1}^{a=w} \left[7.f_1.10^6. \sum^{T_{(t,a)}} Beta(8.8;19) \right],$$

weaned calves T, without access to grazing:

$$Q^{\text{Ts1}}_{(t)} = \sum_{a=w+1}^{a=26} \left[7.f_2.10^6. \sum^{T_{(t,a)}} Beta(8.8;19) \right],$$

weaned calves T, with access to grazing:

$$Q^{\text{Ts2}}_{(t)} = \sum_{a=27}^{a=y} \left[7.f_2.10^6. \sum^{T_{(t,a)}} Beta(8.8;19) \right],$$

young heifers T:

$$Q^{\text{Ty}}_{(t)} = \sum_{a=y+1}^{a=h} \left[7.f_Y.10^6. \sum^{T_{(t,a)}} Beta(8.8;19) \right],$$

heifers T:

$$Q^{\mathrm{TH}}_{(t)} = \sum_{a=h+1}^{a=ad} \left[7.f_A.10^6.\sum^{T_{(t,a)}} Beta(8.8;19) \right],$$

heifers Is:

$$\text{if } \sum_{a=h+1}^{a=ad} \left(Is_{(t,a)} \right) > 0 \;:\quad Q^{\mathrm{ISH}}_{(t)} = \sum_{a=h+1}^{a=ad} \left[7.f_A.10^{(4+10\times\sum^{Is_{(t,a)}} Beta(2.65;17))} \right] \quad \text{else} \quad Q^{\mathrm{ISH}}_{(t)} = 0,$$

cows Is:

$$\text{if } \sum_{i=1}^{i=5} \left(Is_{(t,A_i)} \right) > 0 \;:\quad Q^{\mathrm{IS}}_{(t)} = \sum_{i=1}^{i=5} \left[7.f_A.10^{(4+10\times\sum^{Is_{(t,A_i)}} Beta(2.65;17))} \right] \quad \text{else} \quad Q^{\mathrm{IS}}_{(t)} = 0,$$

heifers Ic:

$$\text{if } \sum_{a=h+1}^{a=ad} \left(Ic_{(t,a)} \right) > 0 \;:\quad Q^{\mathrm{ICH}}_{(t)} = \sum_{a=h+1}^{a=ad} \left[7.f_A.10^{(8+10\times\sum^{Ic_{(t,a)}} Beta(2;17))} \right] \quad \text{else} \quad Q^{\mathrm{ICH}}_{(t)} = 0,$$

cows Ic:

$$\text{if } \sum_{i=1}^{i=5} \left(Ic_{(t,A_i)} \right) > 0 \;:\quad Q^{\mathrm{IC}}_{(t)} = \sum_{i=1}^{i=5} \left[7.f_A.10^{(8+10\times\sum^{Ic_{(t,A_i)}} Beta(2;17))} \right] \quad \text{else} \quad Q^{\mathrm{IC}}_{(t)} = 0.$$

B Parameters ralated to population dynamics

Parameters notation, values and definition are provided in table below.Part of parameters related to population dynamics are specific to each herd, and their values are calibrated from the data (FCID, see table and figure below).

Death rate at birth

Death rate at birth is based on the ratio between the number of animals which died on the day of their birth and the number of animals born during the year:

$$Death\ rate\ at\ birth = \frac{number\ of\ animals\ died\ on\ the\ day\ of\ their\ birth\ during\ the\ year}{number\ of\ all\ births\ during\ the\ year}$$

Death and culling rates

The calculation of death and culling rates is based on the ratio between the number of animals leaving the herd because of self-consumption (SC), culling (C) or death (D), during the year, and the number of presence days of all animals during the same period:

$$Death\ and\ culling\ rates = \frac{number\ of\ animals\ leaving\ the\ herd\ because\ of\ SC,\ C\ or\ D}{number\ of\ presence\ days\ of\ all\ animals}$$

This calculation concerns only the females of dairy or mixed breeds, and is made for different age groups used in the within-herd model: young calves (1 to 2 weeks), unweaned calves (3 weeks to weaning = 10 weeks) , weaned calves + young heifers + heifers (11 to 130 weeks), and cows by age group (1, 2, 3, 4 and 5+).

Parameters for management and population dynamics used in the *Mycobacterium avium subsp. paratuberculosis* (*Map*) infection dynamics model within a structured dairy herd.

Notation	Value	Definition	Source
σ_B	fig. below	Mortality rate of calves at birth	$*$
σ_{c1}	fig. below	Death rate of female calves, weeks 1 and 2	$*$
σ_{c2}	fig. below	Death rate of female calves, weeks 3 to weaning	$*$
σ_{c3}	fig. below	Death rate of heifers from weaning to entry in adult group	$*$
σ_{Ai}	fig. below	Culling rate of cows in adult group i: 1, 2, 3, 4 and above 5	$*$
w	10	Weaning age (weeks)	[43]
y	52	Age when entering the young heifer group (weeks)	
h	91	Age when entering the heifer group (weeks)	†
ad	130	Age when entering the adult group (weeks)	†
τ_{aa}	52	Mean time spent in adult age groups 1 to 4 (weeks)	
b	5	Quantity of colostrum fed to calves (L/day for 3 days)	‡
d	7	Quantity of milk fed to calves after 3 days (L/day/calf)	‡
$prop$	0.85	Proportion of lactating cows	†
ε	25	Quantity of milk or colostrum produced (L/day/cow)	†
f_1	0.5	Quantity of feaces produced by a non-weaned calf (kg/day)	‡
f_2	5.5	Quantity of feaces produced by a weaned calf (kg/day)	‡
f_Y	10	Quantity of feaces produced by a heifer (kg/day)	‡
f_A	30	Quantity of feaces produced by a cow (kg/day)	‡
$Graz$	$[14-26]$	Grazing period (1 being the first week of the year)	‡

$*$ Calibrated on data, herd specific.
† Agricultural statistics.
‡ Based on expert opinion.

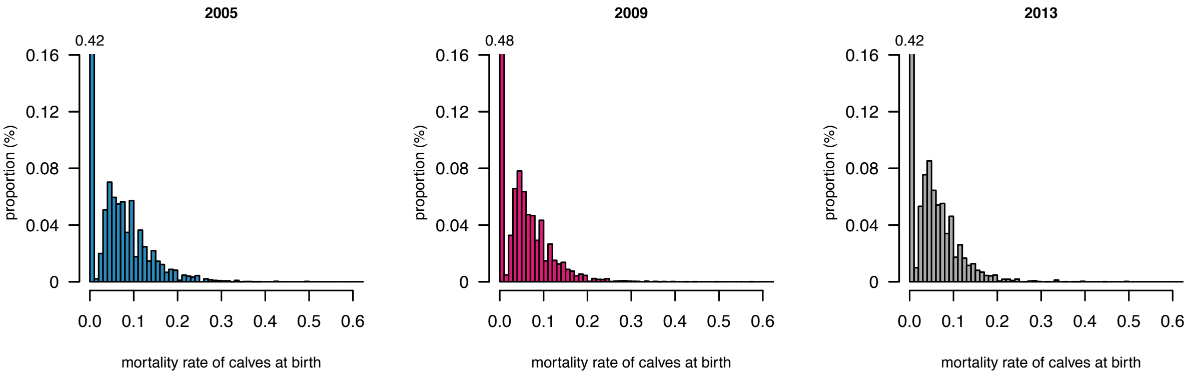

Distributions of the demographic parameters for years 2005, 2009 and 2013, as calibrated from data: distributions of the mortality rates of calves at birth (σ_B).

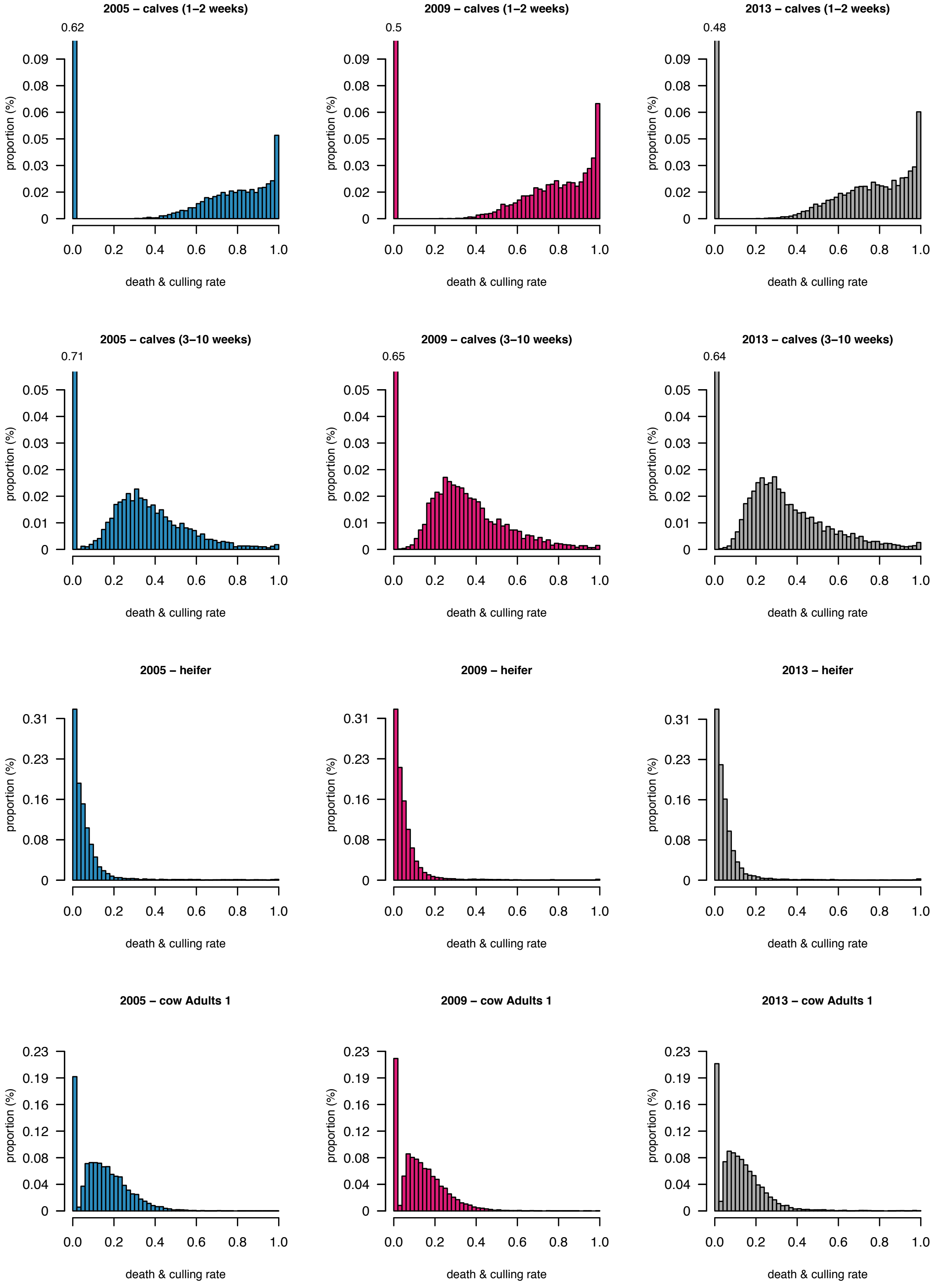

Distributions of the demographic parameters for years 2005, 2009 and 2013, as calibrated from data: distributions of death and culling rates per age group (σ_{c1}, σ_{c2}, σ_{c3} and σ_{A1}).

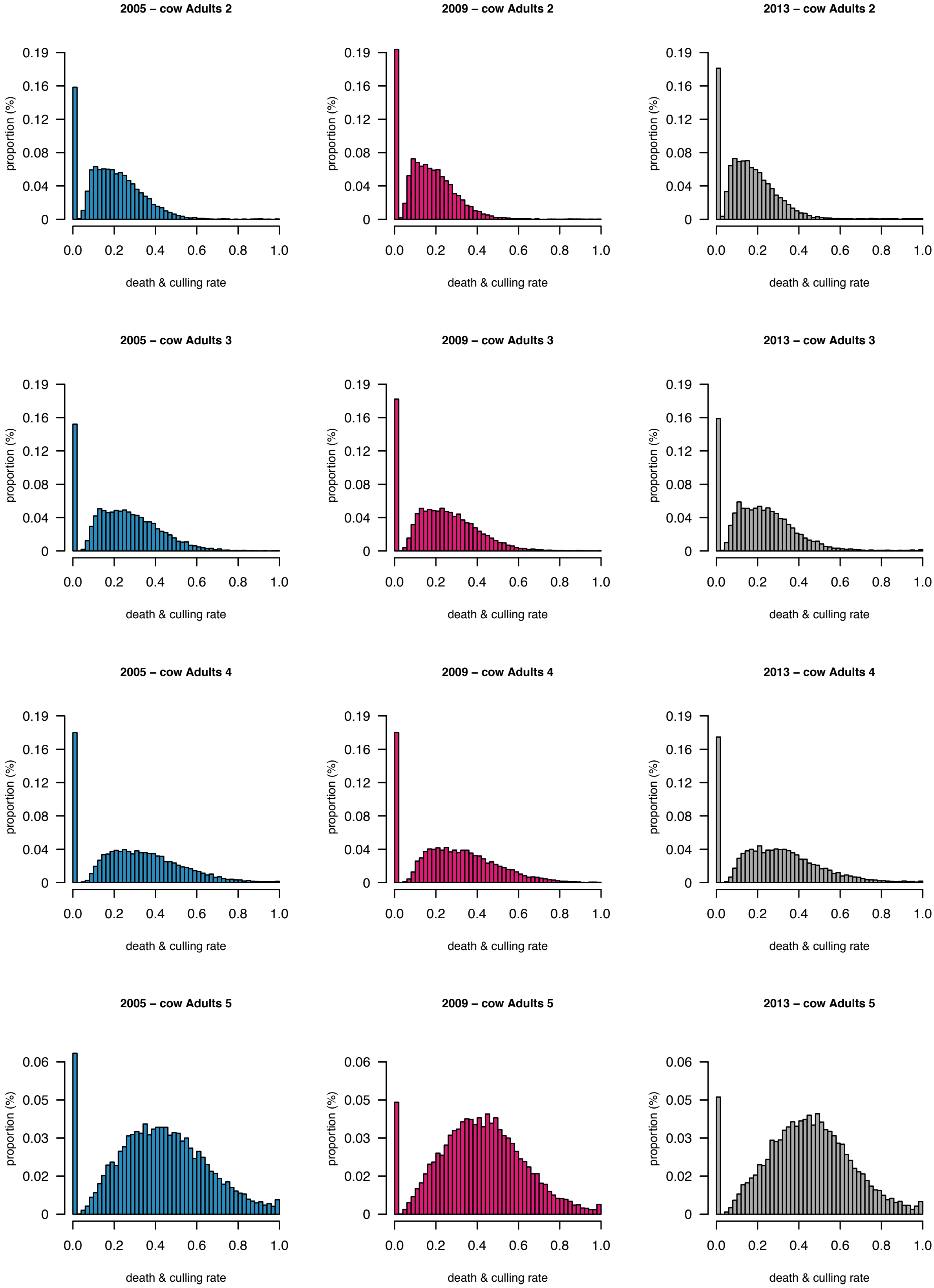

Distributions of the demographic parameters for years 2005, 2009 and 2013, as calibrated from data: distributions of death and culling rates per age group (σ_{A2}, σ_{A3}, σ_{A4} and σ_{A5+}).

C Parameters related to infection dynamics

Parameters for infection and transmission used in the *Mycobacterium avium subsp. paratuberculosis* (*Map*) infection dynamics model within a structured dairy herd (reproduced from [26] - table 2).

Notation	Value	Definition	Source
p_X		Probability of *in utero* transmission for cow in health state X	[20, 24]
	$p_L = 0.149$	X = latently infected (L)	
	$p_{Is} = 0.149$	X = subclinically infected (Is)	
	$p_{Ic} = 0.65$	X = clinically affected (Ic)	
u	52	Maximal age in the susceptible compartment (weeks)	[34, 44]
γ	0.1	Susceptibility follows an exponential decrease : $exp(-\gamma(age-1))$	[19]
v_X		Mean time spent in health state X (weeks)	
	$v_T = 26$	X = transiently infectious (T)	[21]
	$v_L = 52$	X = latently infected (L)	[35, 45]
	$v_{Is} = 104$	X = subclinically infected (Is)	[46]
	$v_{Ic} = 26$	X = clinically affected (Ic)	†
sh_X		Probability of shedding in colostrum or milk for a cow in health state X	[47, 48]
	$sh_L = 0$	X = latently infected (L)	
	$sh_{Is} = 0.4$	X = subclinically infected (Is)	
	$sh_{Ic} = 0.9$	X = clinically affected (Ic)	
α	10^6	*Map* infectious dose	[49]
β_l	$5 \times 10^{-4} \times 7$	Transmission rate if ingestion of an infectious dose (per week)	‡
β_c	$5 \times 10^{-5} \times 7$	Transmission rate if one infectious dose is present in the local environment (per week)	[21]
β_g	$9.5 \times 10^{-7} \times 7$	Transmission rate if one infectious dose is present in the global environment (per week)	[21]
β_o	$5 \times 10^{-6} \times 7$	Transmission rate if one infectious dose is present in the pasture (per week)	‡
g_X		Decrease in milk production for cattle in health state X (per week)	[50]
	$g_L = 1 - 0.08$	X = latently infected (L)	
	$g_{Is} = 1 - 0.11$	X = subclinically infected (Is)	
	$g_{Ic} = 1 - 0.25$	X = clinically affected (Ic)	
μ_k		Removal rate of Map from environment k	[51, 52]
	$\mu_g^{int} = 0.4$	all the environments (per week)	
	$\mu_g^{ext} = 1/14$	all the environments (per week)	
	$\mu_{cp} = 0.17$	collective pens (when empty)	

† Expert opinions.

‡ Parameters' values are assumed.

Summary of published data and assumed distributions of the quantities of *Mycobacterium avium* subsp. *paratuberculosis* (*Map*) shed, depending on the health state (X) and the route of transmission (r) in the *Map* infection dynamics model within a structured dairy herd (reproduced from [26] - table 3).

**Route of transmission (r)**	**Health state (X)**	**Literature**				**Assumed distributions**
		**Minimal value**	**Maximal value**	**Mean value**	**Source**	
Map direct shedding in milk and colostrum (Map/L)	Subclinically infected	2.2×10^4	8.8×10^4	5×10^4	[48]	$10^5 \times Beta(8;8)$
	Clinically affected	-	-	5×10^4	[53]	$10^5 \times Beta(8;8)$
Map indirect shedding in milk and colostrum (faecal contamination) (Map/L)	Subclinically infected	0	2×10^{10}	40	[54, 55]	$1 + 10^3 \times Beta(1;25)$
	Clinically affected	700	2×10^{10}	14×10^4	[54, 55]	$10^{(3+10 \times Beta(50;200))}$
Map shedding in faeces (Map/kg)	Transiently infectious	6×10^4	6.3×10^5	3×10^5	[21]	$10^6 \times Beta(8.8;19)$
	Subclinically infected	10^4	10^{15}	2.6×10^6	[56]	$10^{(4+10 \times Beta(2.65;17))}$
	Clinically affected	10^8	10^{15}	10^{10}	[57, 58]	$10^{(8+10 \times Beta(2;17))}$
