# Supplementary material for: Modelling of paratuberculosis spread between dairy cattle farms at a regional scale
Source: Vet Res. 2015 Sep 25;46:111. doi: 10.1186/s13567-015-0247-3 (PMC4583165; doi:10.1186/s13567-015-0247-3)
Supplement: Additional file 2: — Network characteristics. This file contains graphs representing distributions of herd size and of characteristics of the network describing cattle trade data for Brittany over the period 2005-2013. [file 13567_2015_247_MOESM2_ESM.pdf]

Additional file 2

Network characteristics

This section comprises graphs representing distributions of herd size and of characteristics of the network discribing cattle trade data (FCID) for Brittany and the period 2005-2013.

Node degree measures how many direct contacts a farm has with others in the network. In directed networks, degree can be partitioned into in-degree and out-degree representing the number of potential sources and destinations for disease transmission, respectively.

Node strength measures the number of unit exchange (animals sent and received): out-strength corresponding to outgoing movements and in-strength corresponding to incoming movements.

Polarity represents the ratio between the difference of in and out strengths over their sum. It accounts for the trade behaviors: farms are rather sellers or rather buyers or both at equivalent levels, (as defined in Moslonka-Lefebvre M, Gilligan C, Monod H, Belloc C, Ezanno P, Filipe J, Vergu E: Market analyses of livestock trade networks to inform the prevention of joint economic and epidemiological risk, submitted).

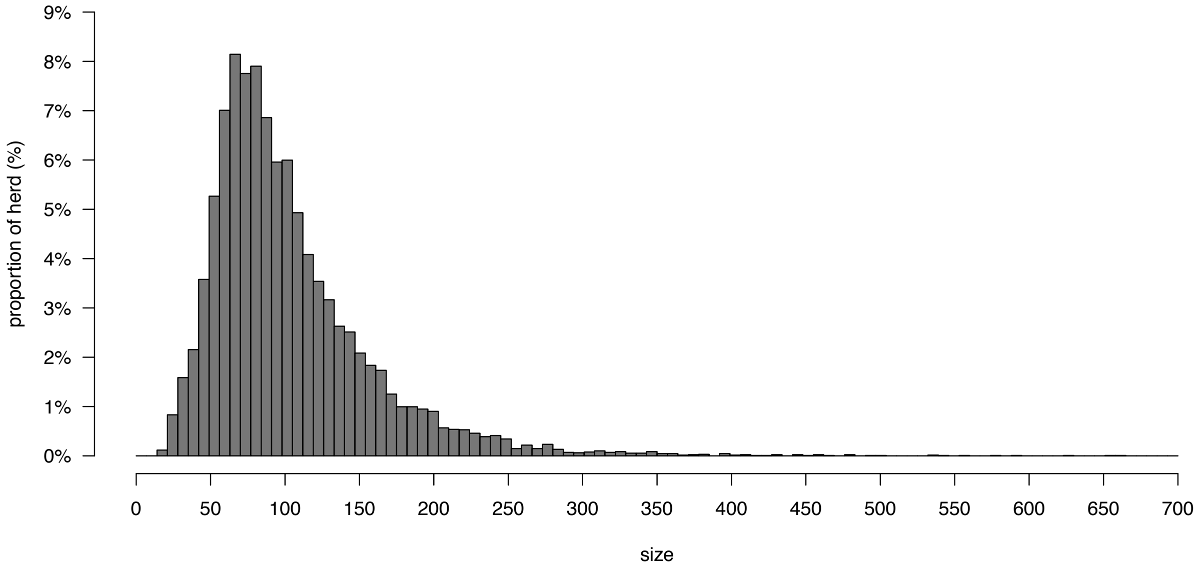

Distributions of the herd sizes of farms in the metapopulation.

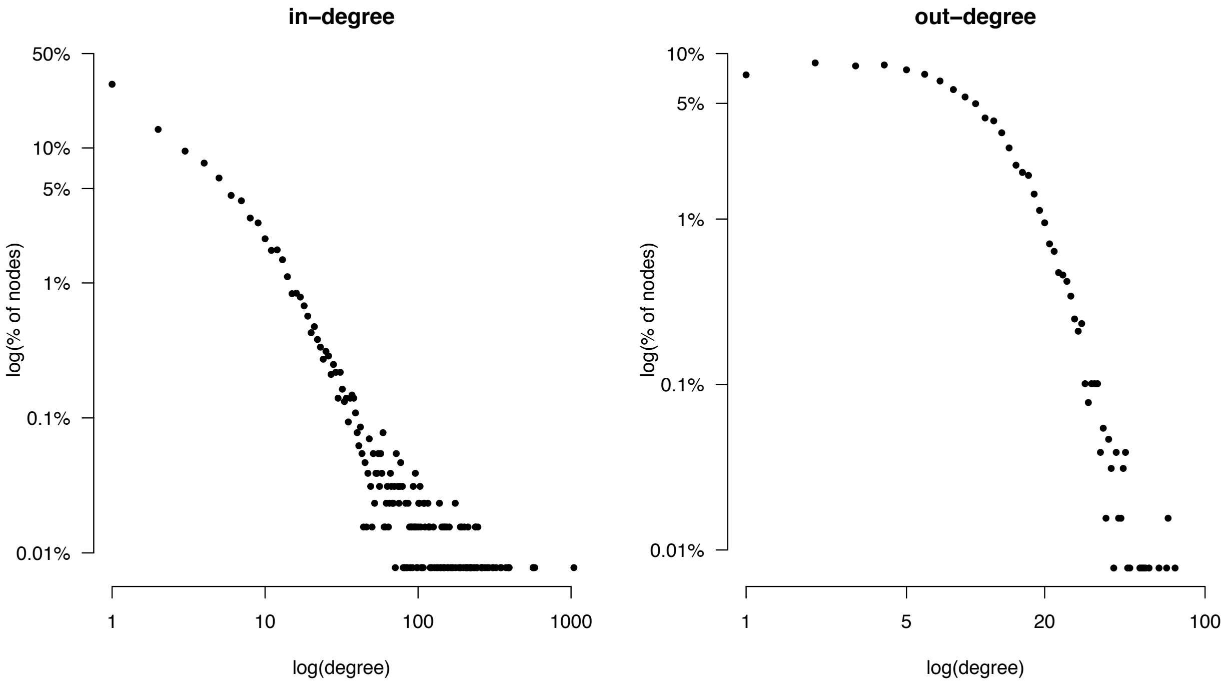

Distributions of the in- (left) and out-degrees (right).

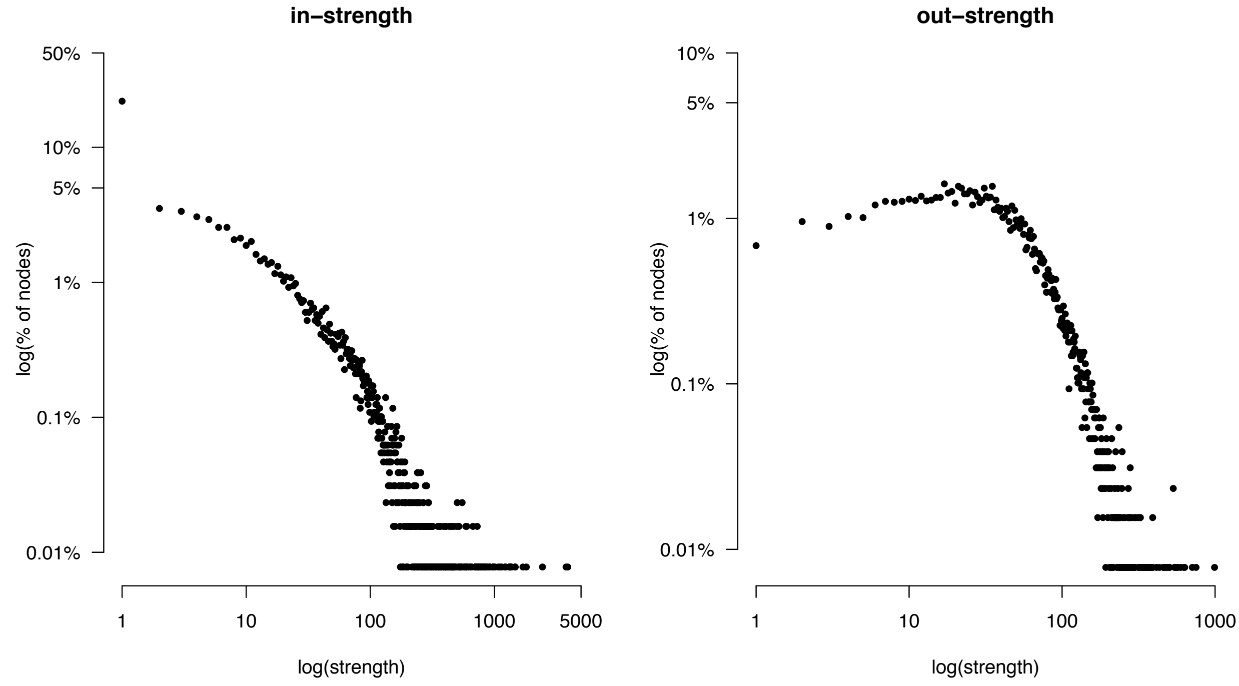

Distributions of the in- (left) and out-strengths (right) taking into account all movements.

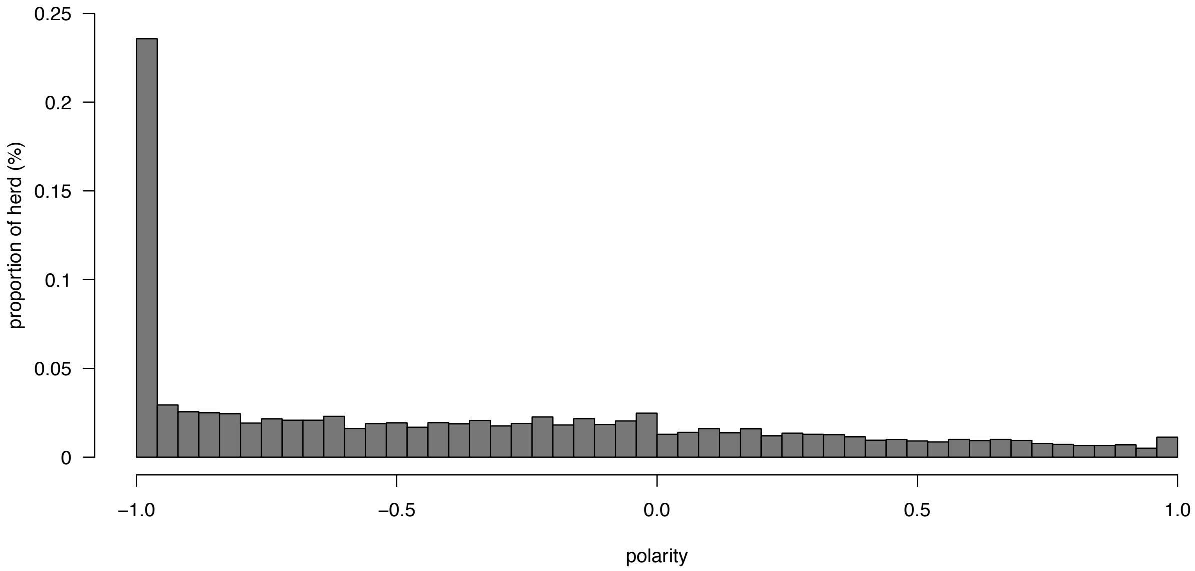

Distributions of the polarity of farms in the metapopulation.
