# Supplementary material for: Modelling of paratuberculosis spread between dairy cattle farms at a regional scale
Source: Vet Res. 2015 Sep 25;46:111. doi: 10.1186/s13567-015-0247-3 (PMC4583165; doi:10.1186/s13567-015-0247-3)
Supplement: Additional file 3: — Population dynamics calibration. This file contains graphs on population dynamics (data versus simulation). [file 13567_2015_247_MOESM3_ESM.pdf]

Additional file 3

Population dynamics calibration

Population dynamics were simulated using herd size and management calibrated on data (explained in additional file 2) and animal movements between herds completely matched observed trade exchanges between herds.

The agreement between observed and predicted herd size over the 9-year period was considered acceptable if there was at most 20% of gap between average predictions and observations on at least seven years among nine.

Preliminary explorations of disease-free population dynamics showed a good agreement between simulated and observed data. Demographic trends were adequately reproduced for 99% of the farms, the model was able to track changes in farm size in most of the cases.

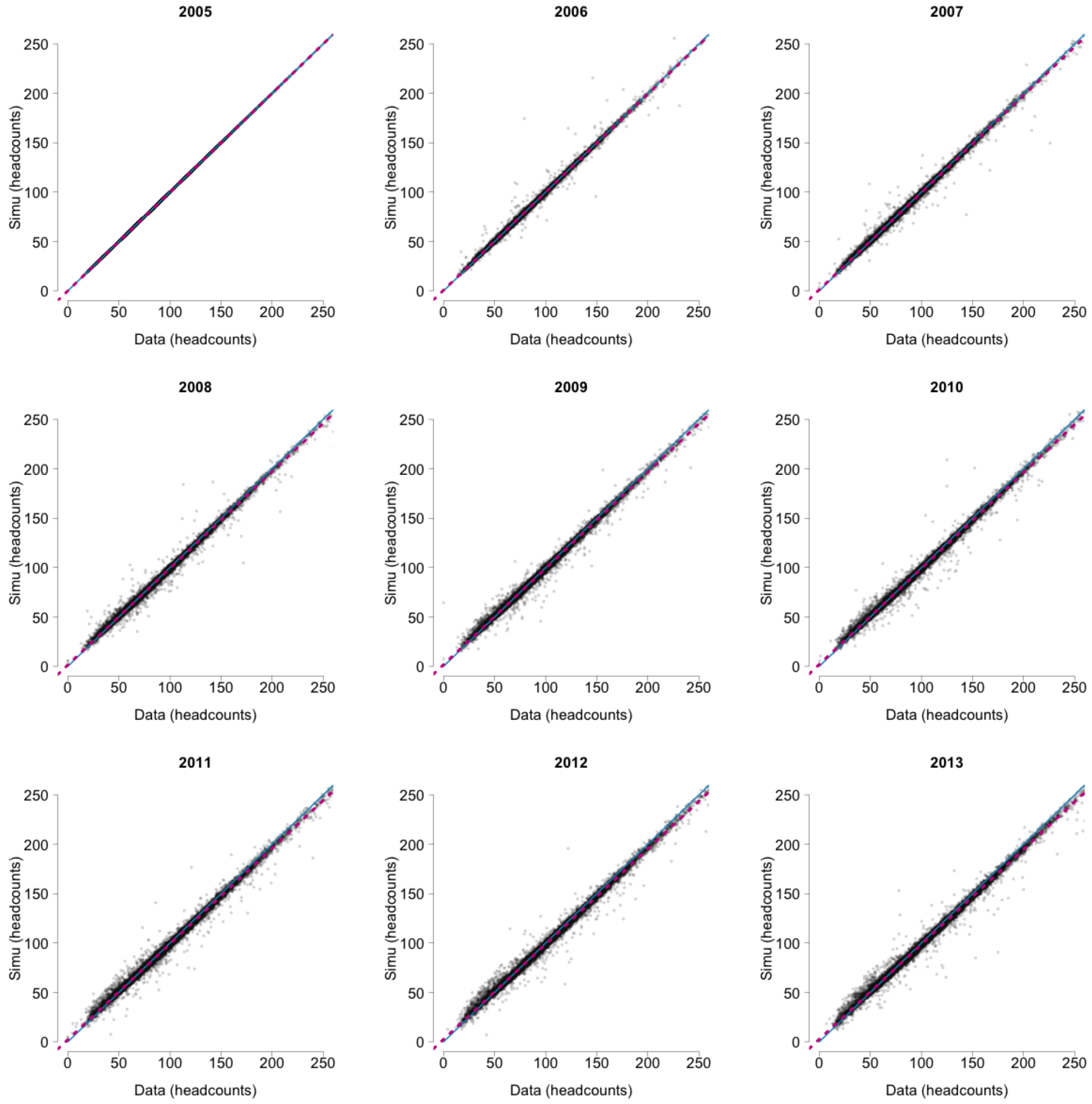

Scatter plot of the population size per year: simulation vs data. Each point corresponds to a herd. Red lines correspond to bisectors, blue lines correspond to simple linear regressions.

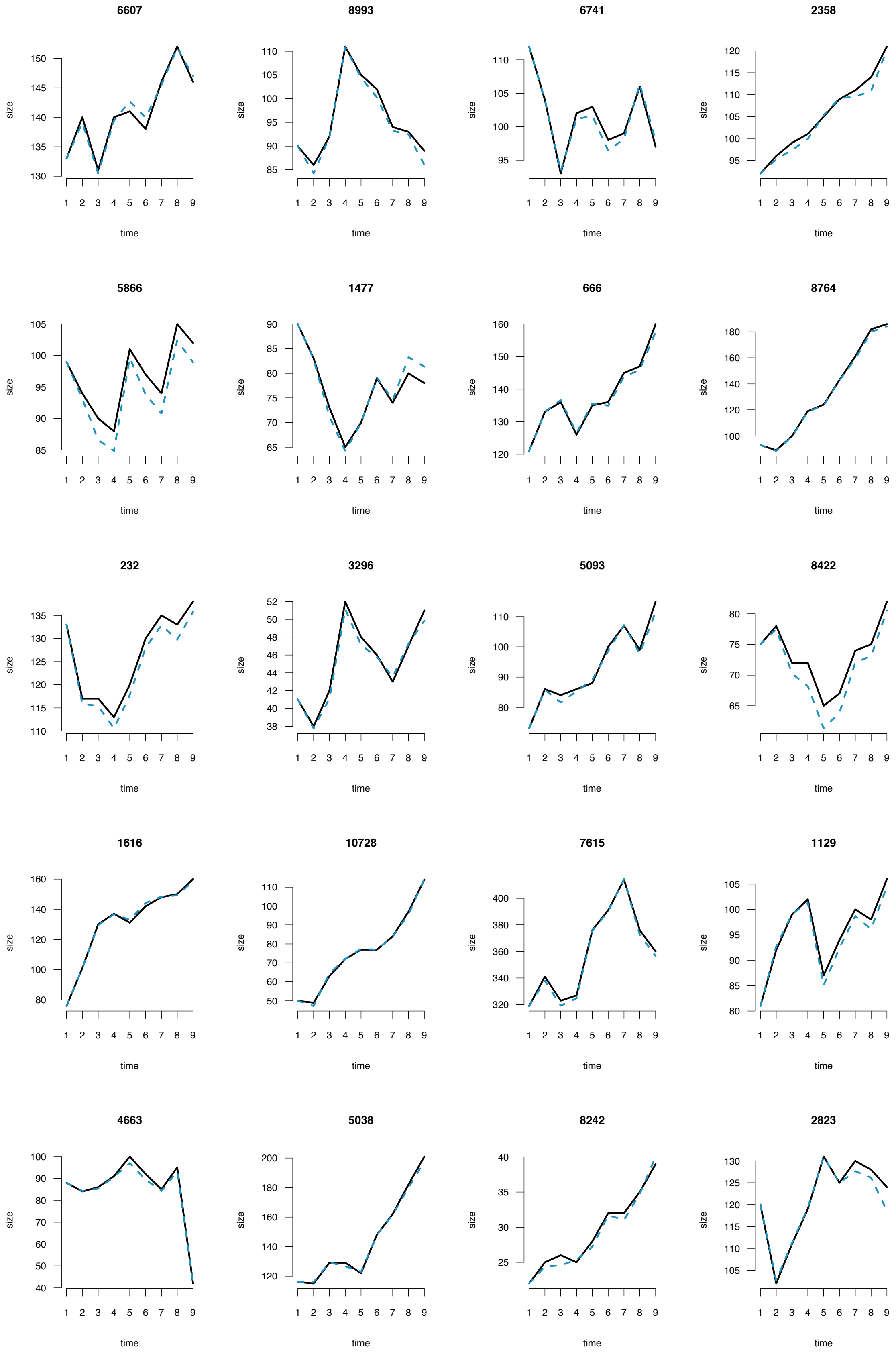

Dynamics of herd size for several herds. Black lines correspond to the data and dashed blue lines correspond to simulated trajectories.
